# Astrocyte-derived HMGB1 compromises the integrity of the blood-brain barrier through the CaM/CaMKII/AQP4 pathway and the protective function of trifluoperazine

**DOI:** 10.3389/fimmu.2026.1852083

**Published:** 2026-06-23

**Authors:** Song-Song Zou, Li-Li Chen, Min Cui

**Affiliations:** 1 Key Laboratory of Exploitation and Study of Distinctive Plants of Sichuan Provincial Education Department, Sichuan University of Arts and Science, Dazhou, China; 2 State Key Laboratory of Agricultural Microbiology, College of Veterinary Medicine, Huazhong Agricultural University, Wuhan, China; 3 Key Laboratory of Preventive Veterinary Medicine in Hubei Province, The Cooperative Innovation Centre for Sustainable Pig Production, Hubei Jiangxia Laboratory, Wuhan, Hubei, China; 4 Key Laboratory of Development of Veterinary Diagnostic Products, Ministry of Agriculture of the People’s Republic of China, Wuhan, Hubei, China; 5 International Research Centre for Animal Disease, Ministry of Science and Technology of the People’s Republic of China, Wuhan, Hubei, China; 6 Dazhou Traditional Chinese Medicine (TCM) Research and Development Centre (R&D), Dazhou, China; 7 Dazhou TCM Industry Incubation Center, Dazhou, China

**Keywords:** blood-brain barrier, HMGB1, JEV, network pharmacology, trifluoperazine

## Abstract

The integrity of the blood–brain barrier (BBB) is crucial for maintaining the function and homeostasis of the central nervous system (CNS), with astrocytes playing a key role in this process. Our study found that infection with the Japanese encephalitis virus (JEV) promoted the translocation of high-mobility group box 1 (HMGB1) from the nucleus to the extracellular space of astrocytes, a process directly associated with BBB disruption. Through bioinformatics analysis, we identified potential targets of encephalitis and constructed a protein–protein interaction (PPI) network. Subsequent functional enrichment analyses, including Gene Ontology (GO) and Kyoto Encyclopedia of Genes and Genomes (KEGG) pathway analyses, highlighted the calcium signaling pathway as an important regulatory mechanism. Evidence from our *in vitro* and *in vivo* model experiments showed that HMGB1 can induce the increase of calcium ions (Ca²^+^) in astrocytes, thereby activating the calcium signaling pathway and promoting the translocation of aquaporin-4 (AQP4) to the plasma membrane, ultimately leading to BBB disruption. We also performed molecular docking and molecular dynamics simulations to determine the binding affinity between trifluoperazine (TFP) and calmodulin (CaM). TFP binds to CaM and blocks the translocation of AQP4 to the plasma membrane, thereby alleviating HMGB1-mediated BBB disruption. Overall, our data indicate that TFP protects BBB integrity through the CaM–CaMKII–AQP4 axis and identifies this pathway as a promising therapeutic target for the clinical treatment of Japanese encephalitis and other central nervous system diseases.

## 1 Background

The blood-brain barrier (BBB) is a dynamic and semipermeable interface in the central nervous system (CNS) that separates the brain from the circulatory system, physically supporting brain homeostasis by controlling the movement of molecules into and out of the CNS. Dysfunction of the BBB is associated with various CNS diseases, including Alzheimer’s disease, stroke, epilepsy, and virus-induced encephalitis, and BBB breakdown exacerbates neurodegeneration, including prion disease (1–5). As a neurotropic virus, Japanese encephalitis virus (JEV) infection causes neuroinflammation and BBB disruption, leading to persistent neurological consequences (6–10). JEV, which is primarily mosquito-borne, with swine as reservoir hosts, has serious implications for human health and animal husbandry economies (8, 9). Our previous study revealed that JEV uses immune cells like “Trojan horses” to enter the CNS, facilitating brain invasion (7). However, the mechanisms underlying JEV-induced BBB breakdown remain unclear.

The BBB comprises cerebral vascular endothelial cells, pericytes, astrocytes, and neurons. Endothelial cells protect the brain from pathogens, toxins, and other hazardous substances, thereby ensuring CNS stability (3, 11, 12). BBB function relies on the crosstalk between CNS cells, particularly vascular endothelial cells, and astrocytes (2). Astrocytes extend processes that wrap around brain microvessels, where they support endothelial tight junctions, regulate ion concentrations, and provide neuronal nutrients (13, 14). *In vitro* studies have demonstrated that astrocytes enhance the strength of endothelial junctions (15, 16). BBB breakdown is a pathological hallmark of severe CNS injury or infection, including cases caused by JEV, WNV, HIV, and SARS-CoV-2 (1, 11, 17). As important components of the BBB, astrocytes drive CNS inflammatory responses and participate critically in CNS diseases (2, 3, 12, 14, 18). During neuroinflammation, astrocyte-derived signals contribute to BBB damage (13, 19); however, the precise mechanisms underlying this process require further elucidation.

High-mobility group B1 (HMGB1) belongs to the HMG protein family and is a highly abundant multifunctional chromatin protein; its biofunction depends on its subcellular location (20). As a damage-associated molecular pattern (DAMP) or alarmin, HMGB1 is released from glial cells upon pathogen invasion (7, 20, 21). Extracellular HMGB1 binds to receptors, including TLR2/4 and RAGE, thereby amplifying inflammation and exacerbating the pathology of sepsis, epilepsy, and stroke (22, 23). Recent studies have indicated that HMGB1 facilitates SARS-CoV-2 invasion and is a potential therapeutic target for COVID-19 (24). Anti-HMGB1 antibodies reduce BBB disruption and neuroinflammation in brain injury and Parkinson’s disease (PD) models, implicating HMGB1 in BBB dysfunction (25); however, the molecular mechanisms underlying HMGB1-mediated BBB disruption remain unclear.

Astrocyte endfeet intimately contact brain microvessels and specifically express aquaporin 4 (AQP4) (26), a key regulator of vascular permeability and neuroinflammation (27–30). This 30 kDa water channel protein forms tetramers that regulate CNS water and ion homeostasis (31). AQP4 deficiency alters BBB morphology, function, and permeability (31, 32). In oxygen–glucose deprivation (OGD) and brain ischemia models, astrocytic ERK/MAPK activation upregulates AQP4, causing BBB disruption (29, 33, 34). The binding of calmodulin (CaM) to AQP4 modulates its translocation to the plasma membrane, contributing to brain oedema (30). Trifluoperazine (TFP) can cross the blood-brain barrier and accumulate in the brain without damaging healthy brain tissue, and may help alleviate neurological symptoms, such as epilepsy and cerebral oedema (30, 35). However, whether TFP can prevent BBB disruption in JEV infections remains unexplored.

In this study, we integrated network pharmacology, molecular docking, and molecular dynamics simulations to investigate the protective effects of TFP on the BBB, and the findings were validated through *in vitro* and *in vivo* experiments. We propose that JEV infection promotes HMGB1 translocation and release from astrocytes, thereby increasing HMGB1 levels in the brain and disrupting the BBB. By combining network pharmacology with GEO database analysis, we identified the specific signaling pathways involved in the ability of TFP to protect the BBB. Additionally, *in vitro* and *in vivo* studies have confirmed the central role of calcium signaling. These findings provide new insights into clinical treatment strategies and offer a scientific basis for repurposing TFP.

## 2 Materials and methods

### 2.1 Mouse and virus

C57BL/6 mice were provided by the Laboratory Animal Center of Huazhong Agricultural University, Wuhan, China. All animal experiments were conducted in accordance with the guidelines of the Animal Care and Use Committee of Huazhong Agricultural University.

The JEV-P3 strain was preserved in our laboratory. A total of 5 × 10^4^ PFU of the viral inoculum was injected into the brains of 1-d-old suckling mice. After euthanization, the mouse’s (symptomatic) brain was collected. Homogenized brains were suspended in Dulbecco’s modified Eagle’s medium (DMEM) at a concentration of 10% (wt/vol). After centrifugation, the supernatant was aliquoted and stored at -80 °C. Baby hamster kidney fibroblast line BHK-21 was used for viral titration via a plaque assay.

All procedures involving anesthesia and euthanasia were meticulously carried out in compliance with the guidelines endorsed by the Institutional Animal Care and Use Committee (IACUC). The study employed specific pathogen-free (SPF) C57BL/6 mice, aged between 6 and 8 weeks. Isoflurane inhalation anesthesia was delivered using a system that included a precision vaporizer, an induction chamber, and a maintenance mask. Anesthesia was initiated with 3–4% isoflurane for a duration of 2–3 minutes until the righting reflex was lost, and was maintained with 1.5–2% isoflurane, balanced with 0.5–1 L/min of oxygen. Continuous monitoring of the pedal withdrawal reflex and respiratory rate was conducted throughout the anesthesia process. For euthanasia, mice were deeply anesthetized with 3–4% isoflurane for induction and 2–2.5% isoflurane for maintenance until a complete loss of consciousness was achieved, as evidenced by the absence of the righting reflex, corneal reflex, and response to noxious stimuli. Euthanasia was then performed via cervical dislocation (C1–C2 separation), with death confirmed by the cessation of respiration, pulse, and all reflexes.

### 2.2 Cell culture

Primary astrocytes were collected from healthy 1-d-old suckling mice and cultured at a density of 1×10^6^ cells/mL in culture medium (DMEM) supplemented with 10% fetal bovine serum (FBS; Gibco, Grand Island, NY, USA), 100 U/mL penicillin, and 100 mg/mL streptomycin sulfate (36).

hBMECs were maintained in our laboratory and grown in DMEM supplemented with 10% FBS and nonessential amino acids (Sigma, Ronkonkoma, NY, USA), minimum essential medium (Sigma, USA), sodium pyruvate (Sigma, USA), 100 U/ml penicillin and 100 mg/mL streptomycin sulfate in a 37 °C incubator with 5% CO_2_. U251 cells were cultured in the laboratory in DMEM supplemented with 10% FBS, 100 U/mL penicillin, and 100 mg/mL streptomycin sulfate in a 37 °C incubator with 5% CO_2_.

### 2.3 Viral infection and recombinant protein treatment of cells

Six- to eight-week-old female mice were randomly divided into two groups: a normal control group and a virus-infected experimental group. In the experimental group (n≥3), the mice were intravenously injected with the virus via the tail vein at a rate of 100 μL (10^5^ PFU/mouse), and tissue samples were collected from days 0– to 6 to detect viral copy numbers in the brain. The control group (n≥3) of mice was injected with 100 μl of serum-free DMEM via the tail vein following the same procedure (6, 36).

After the primary astrocytes, hBMECs, and U251 cells in the culture plate grew to a confluent monolayer, the cells were washed three times with DMEM. The virus or recombinant mouse HMGB1 (ABclonal, Wuhan, China) was inoculated into the wells at an MOI of 1 and incubated at 37 °C for 1.5 h. The cells were then washed three times with DMEM and maintained in DMEM or RPMI-1640 medium supplemented with 2% FBS at 37 °C.

### 2.4 Western blotting

Cells and mouse tissues were lysed in RIPA buffer containing a protease inhibitor cocktail. Protein concentration was determined using a BCA Protein Assay Kit (Beyotime, Shanghai, China). The protein samples were separated by SDS–PAGE using a 12% polyacrylamide gel. Proteins were subsequently transferred to polyvinylidene difluoride membranes (Bio-Rad, Richmond, CA, USA) and blocked for 2 h at room temperature in Tris-buffered saline with Tween 20 (TBST) containing 5% nonfat dry milk. The membranes were subsequently incubated overnight at 4 °C with anti-HMGB1, anti-AQP4, anti-Lamin A/C, anti-Na^+^/K^+^ ATPase, or anti-β-actin monoclonal antibodies (ABclonal, Wuhan, China). After washing with TBST, the membranes were incubated with horseradish peroxidase (HRP-conjugated)-conjugated secondary antibodies. Enhanced chemiluminescence reagents (Bio-Rad, USA) were used to visualize HRP-induced signals.

### 2.5 Intracranial injection

Healthy C57BL/6 mice (female, 6–8 weeks old) were randomly divided into four groups Ten microliters of DMSO, rHMGB1, trifluoperazine (TFP), or rHMGB1+TFP was injected into the mouse brain. For mice treated with rHMGB1+TFP, TFP was injected daily into the abdominal cavity.

### 2.6 BBB permeability measurement *in vivo*

After 48 h (rHMGB1) and 5 days (JEV) of administration of the above reagents, fluorescein sodium (NaFluo, 376 Da) was injected intraperitoneally, and blood was collected from the mice to prepare the serum. PBS was then used to perfuse the mouse brain, and brain tissue supernatant was prepared (6, 36). The average fluorescence density of NaFluo in the serum and brain tissue supernatant was detected using a microplate reader at an excitation wavelength of 488 nm and an emission wavelength of 530 nm. The degree of BBB damage in the mouse body was determined by calculating the fluorescence ratio of NaFluo in the serum and brain tissue supernatant.

### 2.7 H&E and immunofluorescence staining

After deparaffinization, rehydration, and antigen retrieval, the tissue sections were subjected to hematoxylin – and eosin (H&E) staining and imaged via inverted microscopy (Olympus LS, Japan). For immunofluorescence staining, tissue sections or cells were washed three times with PBS for 5 min each and incubated with primary antibody (HMGB1/AQP4, ABclonal, China; GFAP, Proteintech, China) overnight at 4 °C or 1–2 h at 37 °C, followed by incubation with a fluorescent secondary antibody at 37 °C for 1–2 h. Nuclei were stained with DAPI (1:2000). The cell membrane was stained with DiI (Yeasen, China). After washing, images were taken under a laser confocal microscope (Nikon N-STORM, Japan).

### 2.8 Isolation and culture of primary astrocytes

One-day-old suckling mice were anaesthetized with isoflurane and placed on ice for a few minutes until no response was observed (37), after which whole brains were removed and transferred to a Petri dish containing antibiotics and precooled HBSS in a biosafety cabinet. The brainstem and meninges were removed, and the remaining brain tissue was cut into small pieces. The tissue pieces were transferred to a 15 mL centrifuge tube, 6–8 mL of 0.125% trypsin was added, and the tube was digested at 37 °C for 20–30 min with shaking every 5 min. Then, 5 mL of complete culture medium was added to terminate the digestion, 2–3 μL of DNase was added, and the mixture was mixed well and incubated at 37 °C for 5 min. The samples were pipetted 10 times to disaggregate the tissue pieces, and the mixture was allowed to stand for 5 min to allow the tissue blocks to settle. The supernatants containing glial cells were subsequently transferred to a new 15 mL centrifuge tube, the pellets were discarded, and complete medium supplemented with 10% FBS was added for resuspension of the isolated glial cells. The cells were seeded into culture plates of different sizes according to the experimental requirements. The cells were cultured in a CO_2_ incubator at 37 °C. The medium was replaced with fresh medium after 24 h of cultivation, and the medium was replaced with fresh complete medium approximately every 3–4 days. The cells grew to confluence within approximately 10–14 days. After cell purity was determined, the cells were used for experiments.

### 2.9 HMGB1-knockout cells

The sgRNA was designed by inputting the target sequence into the guide design resources provided by Prof. Feng Zhang’s laboratory at MIT and the Broad Institute (https://www.zlab.bio/resources) (October 2020). Multiple pairs of target sequences were designed to ensure successful knockout. Three target sequences were selected based on the website’s design results and ratings: sgRNA-1: GATACTCACGGAGGCCTCTT; sgRNA-2: GAGTATCGCCCAAAAATCAA; and sgRNA-3: AGATATGGCAAAAGCGGACA.

Recombinant plasmids were constructed, including the lentivirus packaging plasmid, PMD2. pMD2. G, psPAX2, and recombinant pHKO-23 were transfected at a ratio of 1:2:3 into HEK293T cells, which were subsequently cultured in 5% FBS at 37 °C and 5% CO_2_ for 60 h. The supernatant was collected and filtered through a 0.45-μm filter membrane. Recombinant lentivirus was used to infect the target cells, which were preliminarily screened with medium containing puromycin, after which single-cell cloning and expansion were performed. PCR was used to measure Cas9 expression, and western blotting was used to measure HMGB1 expression.

### 2.10 Cell cytoplasm, nucleus, and membrane protein extraction

The cells were washed with precooled PBS three times, collected, and centrifuged at 4 °C for 7 min at 1000 × g. The supernatant was discarded, and the cell pellet was resuspended in 100 μL of Buffer A (10 mM HEPES, 10 mM KCl, 0.2 mM EDTA, 3 mM MgCl_2_, 1 mM DTT, 1 mM PMSF, 1% inhibitor cocktail, and 1% NP-40). The samples were incubated on ice for 10 min and then centrifuged at 1,000 × g for 10 min. The resulting supernatant contained cytoplasmic proteins. The pellet was subsequently resuspended in Buffer A and incubated on ice for 10 min, after which the remaining cytoplasmic proteins were discarded after centrifugation. Then, 100 μL of Buffer B (20 mM HEPES, 400 mM NaCl, 1 mM EDTA, 1 mM DTT, and 1% inhibitor cocktail) was added, and the mixture was vortexed for 30 min at 4 °C. The samples were subsequently centrifuged at 14,000 × g for 10 min, after which the resulting supernatant contained nuclear proteins. The samples were divided into aliquots and stored at -80 °C for further experiments.

### 2.11 BBB double-layer transwell model

The production process of the double-layer BBB transwell model was similar to that of the previous single-layer model. In addition, 5 × 10^4^ U251 cells were seeded on the underside of the transwell chamber membrane. After two days of culture, 5 × 10^5^ hBMECs were seeded on the upper side of the transwell chamber membrane. U251 and hBMECs were cocultured for three days in complete medium supplemented with 10% FBS and phenol red-free DMEM in a 37 °C incubator with 5% CO_2_. After the double-layer cells grew to confluence, the TEER value of the model (Millicell, ERS-2) was measured. Transendothelial electrical resistance (TEER) was determined at 37 °C using an ERS2 voltohmmeter (Millipore). After equilibration, raw values were corrected for blank insert background and normalized to membrane surface area (Ω·cm²). Three independent measurements were performed per well at each time point.

The permeability of BBB model was detected using FITC-dextran (10 kDa/70 kDa, Sigma–Aldrich). *In vitro* detection of the HMGB1 knockout double-layer model in U251 (HMGB1^-/-^) and hBMECs (HMGB1^-/-^) was performed as described above.

### 2.12 Intracellular Ca²^+^ detection

The cells were loaded with Fluo-4 AM (Yeasen, China) at a final concentration of 5 μM, and incubated in Hank’s balanced salt solution (HBSS) at 37 °C for 30 min. After washing, cells were treated with rHMGB1 for designated time points (0, 3, 6, 12, 24 h). Fluorescence intensity was quantified using a CytoFLEX (BECKMAN COULTER, USA) flow cytometer, and mean fluorescence intensity (MFI) was analyzed using FlowJo software.

### 2.13 Quantitative real-time PCR analysis

Total RNA was extracted using TRIzol reagent (Invitrogen, USA). One microgram of RNA was used to synthesize cDNA using a ReverTra Ace RT–PCR RT Kit (Toyobo, Osaka, Japan), following the manufacturer’s instructions. SYBR Green (Invitrogen, USA) was used for quantitative real-time PCR using StepOne Plus and StepOne Software v2.2.2 (Applied Biosystems, Foster City, CA, USA). The relative expression of the JEV-C gene was normalized to that of beta-actin. The pcDNA3.0-HA/JEV-C gene plasmid served as a template for generating a standard curve to quantify JEV copy numbers. Primers used for real-time PCR are listed in **Supplementary Table 1**.

### 2.14 Data collection and analysis

This study utilized the Homo sapiens high-throughput gene expression dataset GSE154002 from the NCBI GEO database as the primary research subject. This dataset comprises gene expression profile data from both control and treatment groups (or disease and normal groups). All analyses were conducted using R and relevant Bioconductor packages. The limma package was employed to construct the design matrix, perform linear model fitting, and apply empirical Bayesian testing (eBayes) to calculate each gene’s log2 fold change (logFC), P value, and false discovery rate (FDR). Genes with |logFC| ≥ 1 and P < 0.05 were identified as significantly differentially expressed genes (DEGs). Concurrently, all genes were ranked in descending order by logFC to establish a genome-wide ranked list. Subsequently, gene ontology (GO) enrichment analysis, encompassing biological processes (BP), cellular components (CC), and molecular functions (MF), was conducted using the enrichGO function in the clusterProfiler package. KEGG pathway enrichment analysis was also performed. For both types of analyses, a significance threshold of P < 0.05 was applied for screening. GO and KEGG GSEA analyses were executed to systematically interpret the biological functions and signaling pathways associated with the DEGs, thereby providing a theoretical foundation for subsequent mechanistic studies.

### 2.15 Prediction of potential targets of TFP and encephalitis

The SMILES sequence of TFP was imported into the SwissTargetPrediction database (http://swisstargetprediction.ch/) with the species set to “*Homo sapiens*” to identify potential genetic targets. DrugBank (https://go.drugbank.com/) and Superpred (https://prediction.charite.de/) were used to predict the potential targets of TFP.

The “Encephalitis” database was entered into the GeneCards (https://www.genecards.org/), DisGeNET (https://disgenet.com/), and Online Mendelian Inheritance in Man (OMIM) (https://www.omim.org/) databases to obtain the disease targets and integrate and deduplicate the predicted targets for each disease.

### 2.16 Screening for common potential targets and construction of a protein–protein interaction network

To identify potential targets, we used the Jvenn platform (https://www.bioinformatics.com.cn/static/others/jvenn/index.html). We subsequently explored known and predicted protein–protein interactions via the STRING database, setting the species to “*Homo sapiens*” and applying a “minimum required interaction score” threshold of 0.4 to generate the interaction network of potential target proteins. The network data were exported in TSV format and subsequently used to construct a protein–protein interaction (PPI) network diagram with Cytoscape software (version 3.10.3). Within Cytoscape 3.10.3, we employed the CentiScape 2.2 statistics tool to adjust node size and color intensity based on degree values and modified the thickness of node connections according to combined score values.

### 2.17 Enrichment analysis of gene functions and pathways of potential target proteins

Gene Ontology (GO) and the Kyoto Encyclopedia of Genes and Genomes (KEGG) were employed for enrichment analysis of gene functions and pathways of potential target proteins. The Database for Annotation, Visualization, and Integrated Discovery (DAVID) (https://davidbioinformatics.nih.gov/) was used to conduct GO and KEGG pathway enrichment analyses on the intersecting targets, with “*Homo sapiens*” as the species of interest. This analysis aimed to obtain enrichment results for key target proteins related to ATBC and neurodegenerative diseases in terms of gene function and signaling pathways. Based on the quantity of enriched genes, the top five terms for each GO category (biological process, cellular component, and molecular function) and the top 20 KEGG pathways were selected and sorted by the number of enriched genes (https://cnsknowall.com/).

### 2.18 Molecular docking and molecular dynamics simulation studies

The crystal structures of these core targets were obtained from the UniProt database (http://www.uniprot.org/), whereas the chemical structure of the TFP compound in SDF format was obtained from the PubChem database. Preprocessing steps, including file format conversion, hydrogen addition, water removal, and ligand stripping, were performed using Open Babel 2.3.2, PyMOL 2.2.0, and AutoDock Vina software. Discovery Studio 2019 visualization software was then used to analyze the details of the ligand–receptor interactions and to create 2D and 3D interaction diagrams.

For the molecular dynamics (MD) simulations, GROMACS 2022.4 software was employed to conduct full-atom MD simulations of protein–ligand complexes obtained from molecular docking. The protein components were parameterized with the Amber14SB force field, and topology files for small molecules were generated using the ACPYPE and Antechamber programs. The protein–ligand complex was placed within a cubic solvation box, ensuring a minimum distance of 1 nm from the edges of the system to the complex. The TIP3P water model was utilized, and the system was neutralized by adding an appropriate amount of sodium and chloride ions. Energy minimization was performed using the steepest descent method, followed by temperature equilibration under the NVT ensemble and pressure equilibration under the NPT ensemble to maintain a system temperature of 300 K and a pressure of 101.325 kPa. Each equilibrated system underwent 100 ns of molecular dynamics simulation at 300 K, yielding 10,000 frames of simulation trajectories. These trajectory files were then used for a detailed analysis of key parameters, including RMSD, RMSF, SASA, RG, and hydrogen bonding.

### 2.19 Ethics statement

All animal experiments were approved by the Research Ethics Committee of Huazhong Agricultural University, Hubei, China (HZAUMO-2019-060) and were performed in accordance with the Guidelines for the Care and Use of Laboratory Animals of the Research Ethics Committee, Huazhong Agricultural University, Hubei, China.

### 2.20 Statistical analysis

All experiments were repeated at least three times. Data are expressed as the means ± SEM. Data were analyzed using the Student’s t-test or one-way analysis of variance, followed by Tukey’s *post hoc* test. Graphs were generated and analyzed using GraphPad Prism software (v7.0; GraphPad, La Jolla, CA, USA).

## 3 Results

### 3.1 JEV infection induces HMGB1 translocation and release in astrocytes

**Figure 1:**
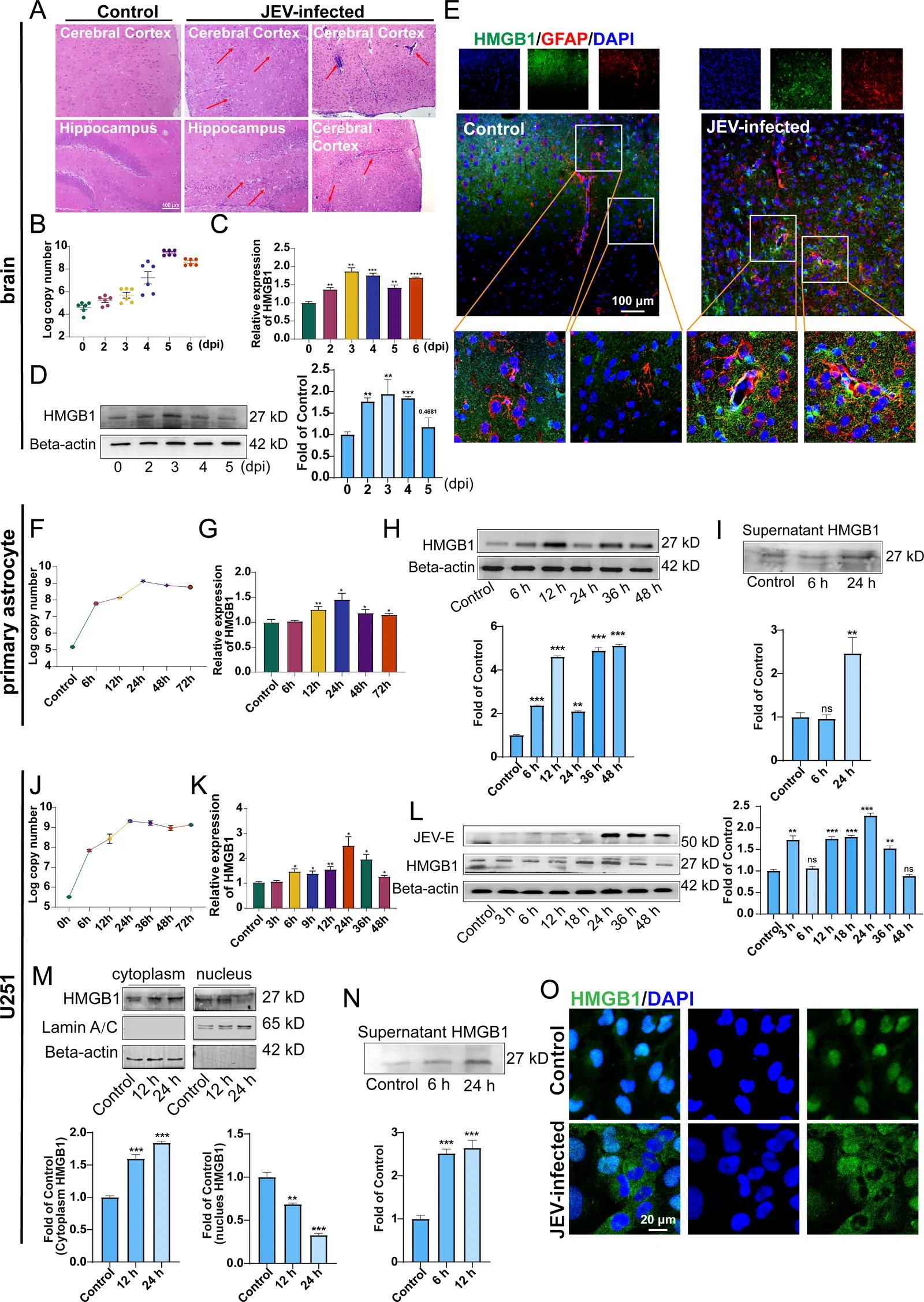
JEV infection induces HMGB1 translocation and release from astrocytes. **(A)** Neuropathological effects of JEV infection (5 dpi) were observed via H&E staining of mouse brains. Cytoplasmic vacuolation, nuclear contraction, distortion, and perivascular cuffs are indicated by red arrowheads. All brain specimens used in this study were harvested from age-matched mice, and all experimental analyses were performed using anatomically consistent coronal brain sections to guarantee sample uniformity. **(B)** Virus copy number detection: The JEV-C gene was measured via real-time PCR in the brain following JEV-P3 (10^5^ PFU) injection. HMGB1 expression was also evaluated by real-time PCR **(C)** and western blotting **(D)**, with the corresponding statistical analysis shown to the right. **(E)** Immunofluorescence images of JEV-infected mouse brains stained for DNA (blue, DAPI), GFAP (red), and HMGB1 (green). A zoomed-in image illustrates the HMGB1 expression pattern in the mouse brain. **(F)** Viral copy number detected at various time points (0–72 h) in primary astrocytes. HMGB1 expression was also determined by real-time PCR **(G)** and western blotting **(H)**, with the corresponding statistical analysis provided at the bottom. **(I)** Supernatants from primary astrocyte cultures were analyzed for HMGB1 release following JEV infection. Statistical analysis is shown at the bottom. **(J)** Viral copy number detected from 0–72 h in U251 cells. HMGB1 expression was confirmed by real-time PCR **(K)** and western blotting **(L)** with the corresponding statistical analysis provided at the bottom, and JEV-E protein was detected by western blotting **(L)**. **(M)** Cytoplasmic and nuclear protein fractions were extracted to detect HMGB1 expression via western blotting, with the statistical analysis provided below. **(N)** Astrocyte (U251) culture supernatant was collected to measure the released HMGB1 after JEV infection, with statistical analysis presented at the bottom. **(O)** Confocal microscopy was used to assess HMGB1 localization in U251 cells; HMGB1 (green) and nuclei (blue) were stained. The scale bars of **(D, E)**, and **(O)** are 100 μm. All experiments were repeated at least three times. The blue histogram displays the results of the statistical analysis of the western blot data. Data are expressed as the means ± SEM. Statistical significance is denoted as *p < 0.05, **p < 0.01, and ***p < 0.001. ns, non significant.

Viral replication in the brain was measured using real-time PCR after JEV infection. Histopathological analysis revealed JEV-induced cytoplasmic vacuolation, nuclear pyknosis, and brain tissue distortion, with immune cell recruitment to the perivascular and parenchymal regions forming perivascular cuffs (**Figure 1A**). The virus was detectable at 2 dpi and peaked at 5–6 dpi (**Figure 1B**). Concurrently, HMGB1 expression increased at 2 dpi and peaked at 3 dpi at both the mRNA (**Figure 1C**) and protein (**Figure 1D**) levels. Further immunofluorescence staining confirmed astrocyte activation and tight encirclement of the brain microvasculature (**Figure 1E**), along with the upregulation of HMGB1 in astrocytes.

We isolated primary astrocytes from the brains of newborn mice and subjected them to JEV infection (**Figure 1F**). JEV infection increased HMGB1 mRNA and protein levels (**Figures 1G, H**) and HMGB1 release (**Figure 1I**) in astrocytes. In U251 cells, JEV infection upregulated HMGB1 mRNA, which peaked at 24 h (**Figure 1J**), and protein expression, which peaked at 18 h (**Figures 1L, M**). Because nuclear-to-cytoplasmic translocation of HMGB1 precedes its release, we assessed the subcellular localization of HMGB1. JEV infection decreased nuclear HMGB1 levels and increased cytoplasmic HMGB1 levels (**Figure 1M**). JEV infection consistently promoted HMGB1 release from astrocytes, as evidenced by the increased HMGB1 levels in the culture supernatant (**Figure 1N**). Neurons may be another source of HMGB1 synthesis (data not shown). These results demonstrate that JEV infection induces HMGB1 release from astrocytes.

### 3.2 HMGB1 increases BBB permeability through positive feedback

Extracellular HMGB1 amplifies the inflammatory response through a potential positive feedback loop (20, 38). Recombinant HMGB1 was found to increase intracellular HMGB1 levels in both primary astrocytes (**Supplementary Figures 1A, B**) and U251 cells (**Supplementary Figures 2A, B**). Concurrently, the expression of HMGB1 receptors, TLR2/4 and RAGE, was elevated in primary astrocytes (**Supplementary Figures 1C–E**) and U251 cells (**Supplementary Figures 2C–E**) following HMGB1 administration.

**Figure 2:**
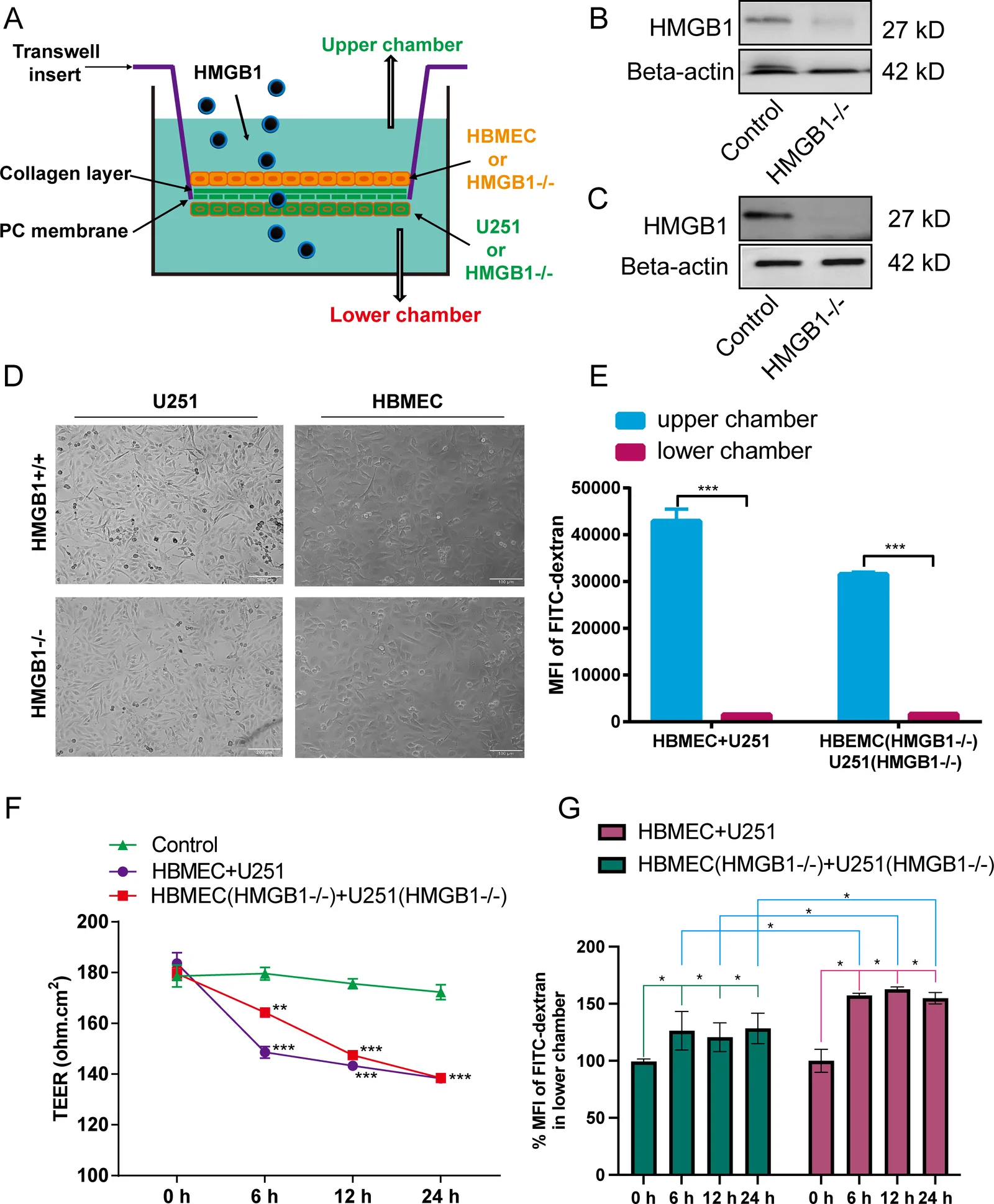
HMGB1 compromises BBB integrity. **(A)** Schematic diagram of the *in vitro* BBB double-layer model, featuring HMGB1 expression (HBMEC + U251) and HMGB1 deletion (HBMEC (HMGB1-/-) + U251 (HMGB1-/-)) models. HMGB1 was knocked out in U251 cells **(B)** and HBMECs **(C)** via CRISPR-Cas9 and verified by western blotting. **(D)** Representative images of cell morphology, including U251 cells, HMGB1-knockout U251 cells (HBMECs-/-), HBMECs, and HMGB1-knockout HBMECs (HBMECs-/-). **(E)** The permeability of the BBB double-layer model was assessed by measuring the MFI of FITC-dextran in the upper and lower chambers. **(F)** The TEER values of the BBB bilayer models were measured at different time points after rHMGB1 treatment (0, 6, 12, and 24 h) and compared with the control rHMGB1-free model. **(G)** FITC-dextran infiltration in the lower chamber after rHMGB1 treatment (0, 6, 12, and 24 h) compared with the control group (free rHMGB1). The scale bar for **(D)** is 200 μm. All experiments were repeated at least three times. Data are expressed as the means ± SEM. Statistical significance is denoted as **p < 0.01 and ***p < 0.001.

To investigate the influence of HMGB1 on blood-brain barrier (BBB) integrity, a bilayer BBB model was established by coculturing hBMECs and U251 cells (**Figure 2A**). Through the application of CRISPR-Cas9 technology, we successfully created HMGB1-knockout hBMECs (HMGB1-/-) and U251 cells (HMGB1-/-) (**Figures 2B, C**). It is noteworthy that the absence of HMGB1 did not significantly alter the morphology or proliferation rates of hBMECs or U251 cells (**Figure 2D**).

In the hBMEC-U251 transwell model, the detection of minimal FITC-dextran leakage in the lower chamber (**Figure 2E**) validates the model’s effectiveness for examining BBB permeability. Similarly, the HMGB1-/- hBMEC-U251 (HMGB1-/-) Transwell model demonstrated preserved BBB integrity (**Figure 2E**). Conversely, the introduction of recombinant HMGB1 (rHMGB1) led to a decrease in the transendothelial electrical resistance (TEER) of the bilayer model, signifying a time-dependent increase in permeability (**Figures 2F, G**, blue lines). Importantly, the knockout of HMGB1 reduced the extent of rHMGB1-induced damage, as indicated by a modest decline in TEER and FITC-dextran leakage (**Figures 2F, G**, red lines). These results, which exclude the intracellular positive feedback effect of HMGB1, further verify that extracellular HMGB1 participates in BBB disruption.

### 3.3 Calcium signaling pathway is significantly enriched

**Figure 3:**
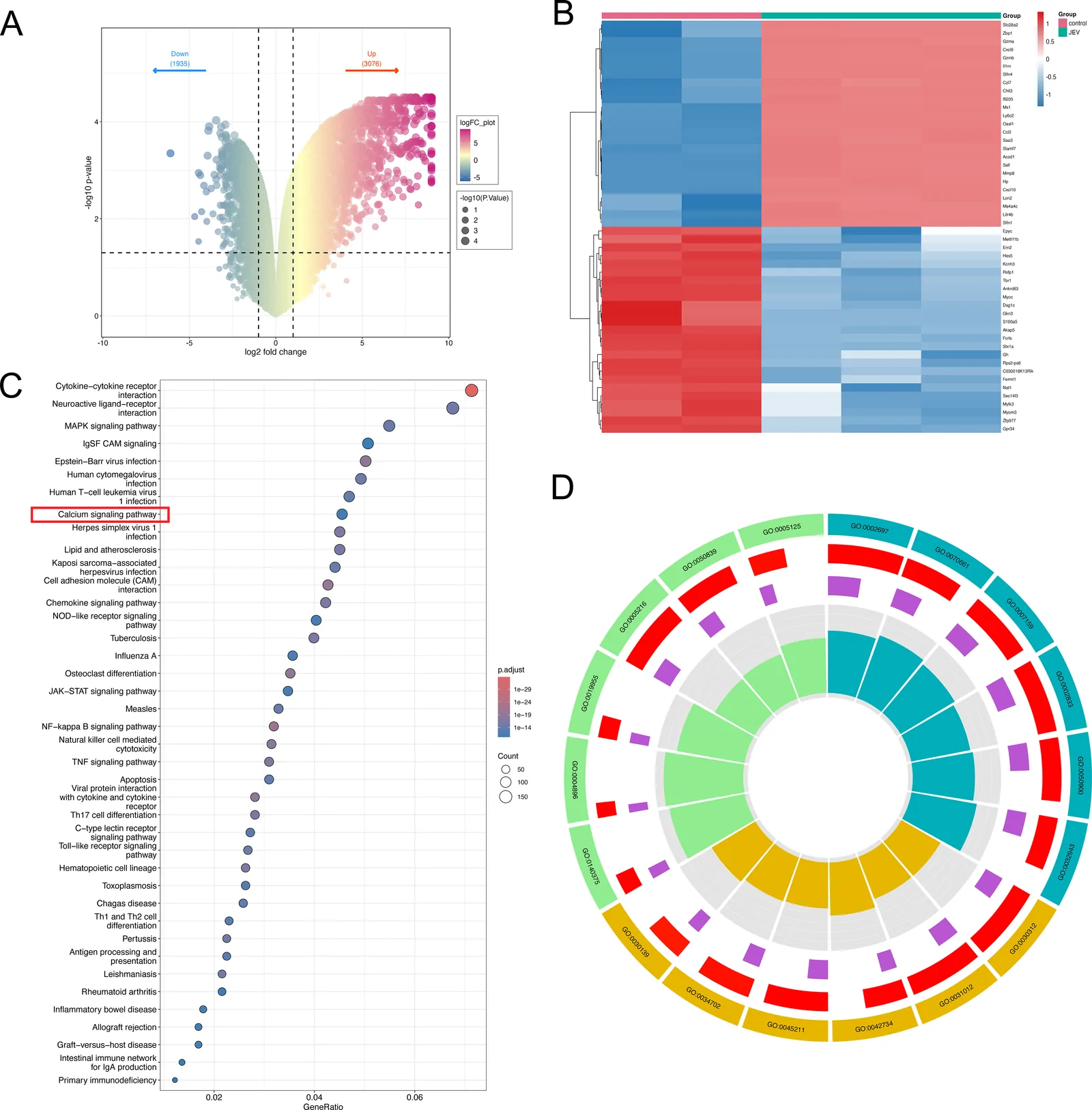
Transcriptome analysis of differentially expressed genes (DEGs) and functional enrichment between JEV-infected and control. **(A)** Volcano plot of DEGs. Red: significantly up-regulated genes, green: significantly down-regulated genes, black: non-differentially expressed genes. **(B)** Heatmap of DEGs, showing the expression pattern clustering of samples and genes (red: high expression, blue: low expression). **(C)** Bubble plot of KEGG pathway enrichment, highlighting the significantly enriched calcium signaling pathway (red box). **(D)** Circle plot of GO functional enrichment, integrating pathway categories, gene numbers, and expression changes.

To elucidate the differences in gene expression and regulatory mechanisms following JEV infection, transcriptome sequencing data were normalized, and differentially expressed genes (DEGs) were identified using thresholds of log2FC ≥1 and P<0.05. The reliability of the data and the specificity of intergroup expression were validated through the use of volcano plots (**Figure 3A**), MA plots (**Supplementary Figure 3A**), and clustered heatmaps (**Figure 3B**). A total of 5,011 DEGs were identified, comprising 1,935 upregulated and 3,076 downregulated genes (**Figure 3A**, **Supplementary Figure 3B**). KEGG pathway enrichment analysis of the DEGs (**Figure 3C**) identified the top 40 significantly enriched pathways. The size of each bubble represents the number of enriched genes, while the color indicates the enrichment significance (-log10 P-value). The analysis revealed that the DEGs were predominantly enriched in biological pathways associated with cell proliferation, differentiation, and immune regulation, including cytokine–cytokine receptor interaction, PI3K-Akt signaling pathway, MAPK signaling pathway, and calcium signaling pathway (**Figure 3C**). Further examination using Gene Ontology (GO) functional enrichment analysis (**Figure 3D**) demonstrated that the top five significantly enriched entries in each functional category were highly significant. Among the biological processes, the regulation of immune effector processes was central, with other immune-related processes, such as leukocyte proliferation and leukocyte adhesion, also highlighted. In terms of cellular components, enrichment was observed in envelope structures, the extracellular matrix, and pre- and postsynaptic membranes. Molecular functions were concentrated on immune receptor activity, cytokine receptor activity/binding, and monoatomic ion channel activity. The KEGG database-based calcium signaling pathway diagram (**Supplementary Figures 3C, D**) illustrates the expression states of key genes in this pathway and the changes in expression patterns of calcium signaling pathway genes after JEV infection. Compared to the control group, several key node genes of the calcium signaling pathway, including PLC, IP3R, RYR, and CaMK, were significantly upregulated in the experimental group (red box), suggesting that the treatment factor may regulate downstream biological processes, such as cell proliferation, apoptosis, and gene transcription, by activating the calcium signaling pathway. Through transcriptome sequencing analysis, a total of 5,011 DEGs were identified, and KEGG enrichment analysis indicated that the calcium signaling pathway is a key regulatory pathway. The expression of several core genes in the calcium signaling pathway changed significantly, suggesting that this pathway may play an important mediating role in the biological effects induced by the treatment factor.

### 3.4 HMGB1 drives AQP4 subcellular translocation in astrocytes via Ca²^+^ signaling

**Figure 4:**
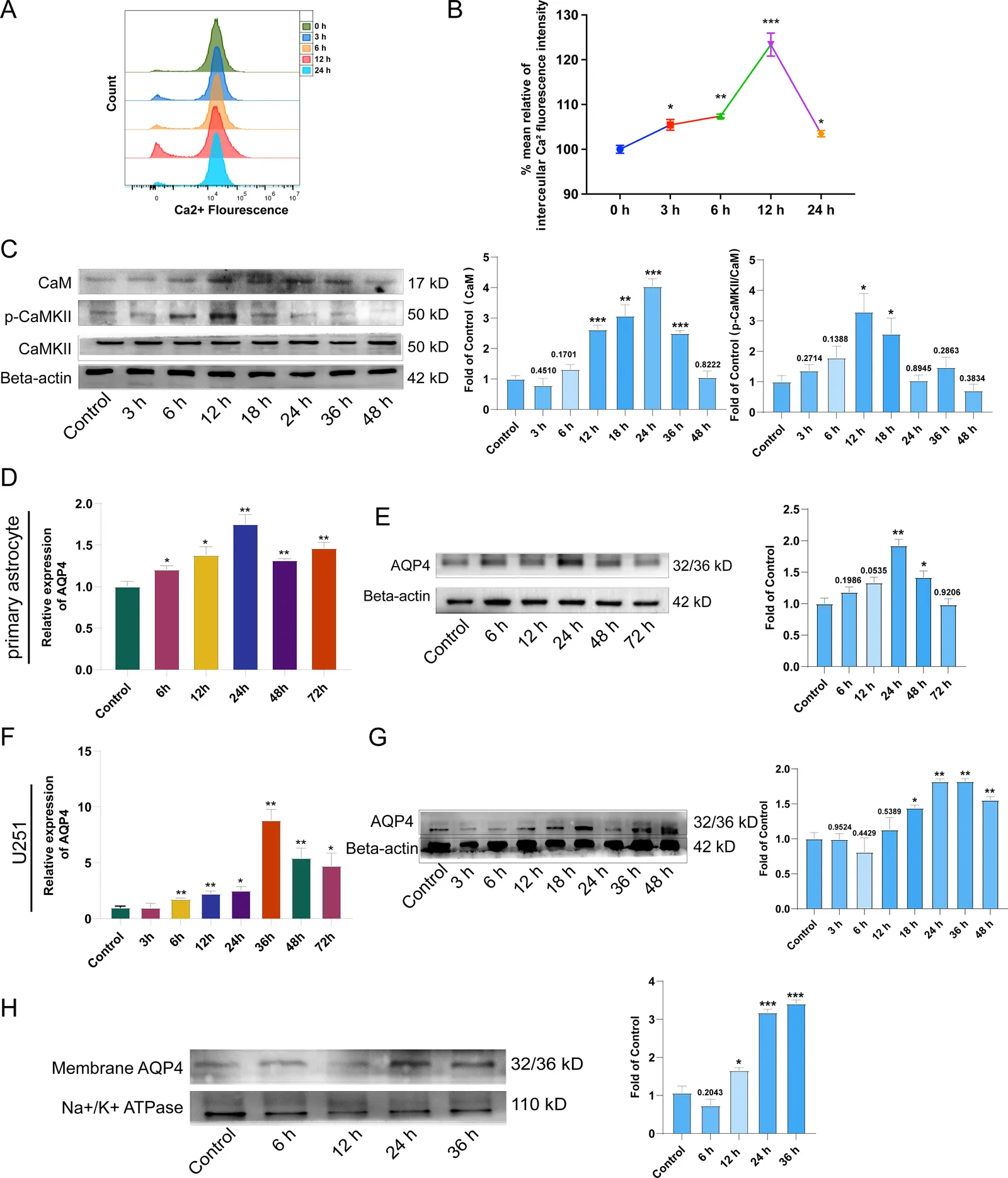
HMGB1 enhances the subcellular translocation of AQP4 in astrocytes. During the addition of rHMGB1, the Fluo-4 AM calcium ion probe was used in conjunction with a flow cytometer. **(A)** Representative flow cytometry histograms showing Fluo-4 AM fluorescence intensity in U251 cells treated with rHMGB1 for the indicated time points (0–24 h). The x-axis represents fluorescence intensity (log scale), and the y-axis represents cell count. **(B)** Quantification of relative mean fluorescence intensity (MFI) ratio of intracellular calcium in U251 cells. Relative intracellular calcium (Fluo-4 AM) MFI in U251 cells at 3 h, 6 h, 12 h, and 24 h following rHMGB1 treatment, normalized to the 0 h control. **(C)** The expression and phosphorylation levels of CaM and CaMKII in U251 cells were determined by western blotting. Real-time PCR **(D, E** and western blotting **(F, G)** were used to measure the mRNA and protein levels of AQP4 in primary astrocytes and U251 cells after rHMGB1 treatment. **(H)** Cell membrane proteins were extracted, and the expression level of AQP4 on the cell membrane was detected by western blotting. All experiments were repeated at least three times. The blue histogram shows the results of the statistical analysis of the western blot data. These data are expressed as the means ± SEMs. *p < 0.05, **p < 0.01, and ***p < 0.001.

Since CaM regulates AQP4 membrane translocation (30), we investigated whether HMGB1 affects intracellular Ca²^+^ dynamics in astrocytes. U251 cells loaded with the Ca²^+^-sensitive fluorescent indicator Fluo-4 AM were treated with rHMGB1, and intracellular Ca²^+^-associated fluorescence was monitored by flow cytometry over a 24-hour time course. As shown in **Figure 4A** and quantified in **Figure 4B**, rHMGB1 induced a time-dependent increase in Fluo-4 fluorescence intensity, with a progressive rightward shift of the fluorescence peak from 0 to 24 h. Notably, a bimodal distribution emerged at 12 h, suggesting cellular heterogeneity in the Ca²^+^ response (**Figure 4A**).

Concurrently, Western blot analysis revealed that rHMGB1 upregulated total CaM expression and enhanced phosphorylation of calcium/calmodulin-dependent protein kinase II (CaMKII) in U251 cells (**Figure 4C**), consistent with activation of the Ca²^+^/CaM/CaMKII signaling axis. In parallel experiments, rHMGB1 treatment increased AQP4 mRNA and total protein levels in both primary mouse astrocytes (**Figures 4D, E**) and U251 cells (**Figures 4F, G**). Moreover, membrane protein extraction and Western blotting demonstrated that rHMGB1 enhanced AQP4 localization to the plasma membrane in U25 cells (**Figure 4H**). Collectively, these results demonstrate that rHMGB1 treatment is associated with increased intracellular Ca²^+^-associated fluorescence, upregulation of CaM/CaMKII signaling, and enhanced AQP4 expression and membrane localization in astrocytes.

### 3.5 Induction of AQP4 expression by JEV infection

Subsequent analyses revealed that both mRNA and protein levels of AQP4 were significantly elevated in mouse brains following JEV infection (**Supplementary Figures 4A, B**). Similarly, an increase in AQP4 expression was observed in primary mouse astrocytes (**Supplementary Figures 4C, D**) and U251 cells (**Supplementary Figures 4E, F**) *in vitro* upon JEV infection. Immunofluorescence staining demonstrated that JEV infection activated astrocytes in the mouse brain, which were closely associated with blood vessels and increases in AQP4 translocation to the BBB and total AQP4 levels (**Supplementary Figures 4G–I**). These findings suggest that AQP4 is responsive to JEV infection and is upregulated in activated astrocytes. In conclusion, JEV infection and extracellular HMGB1 can enhance AQP4 expression, potentially contributing to the disruption of the blood-brain barrier (BBB).

### 3.6 Network pharmacology links encephalitis and TFP

**Figure 5:**
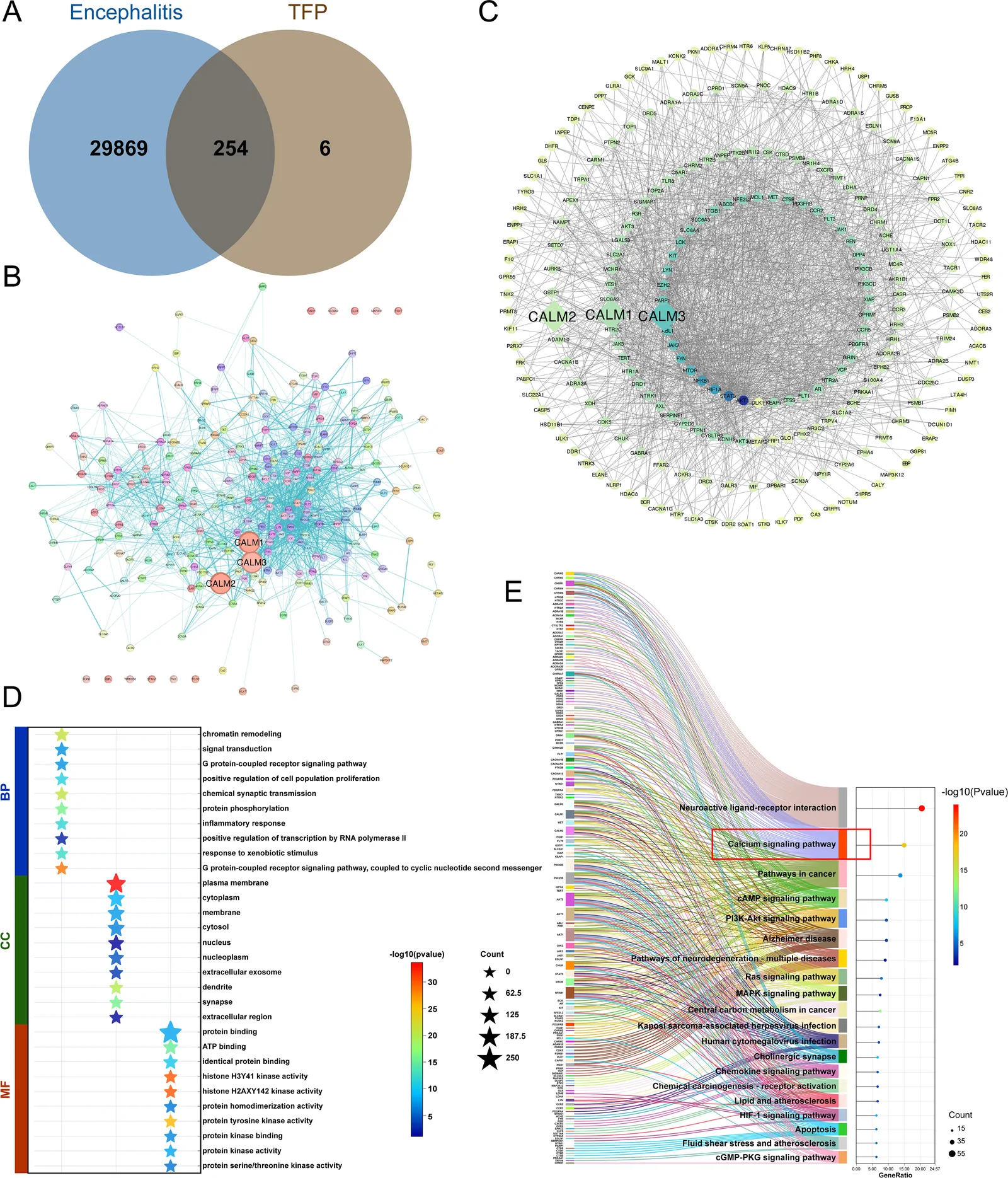
Potential targets and enrichment analysis. **(A)** Venn diagram showing the overlapping targets between encephalitis and TFP (**Supplementary Table 2**). **(B)** A protein–protein interaction (PPI) network was constructed and analyzed using the STRING database. **(C)** Cytoscape was used to visualize and identify the targets associated with TFP in CNS diseases. **(D)** Construction of enriched ontology cluster networks for the intersection targets derived from the GO analysis. **(E)** Sankey diagram illustrating the results of the KEGG enrichment analysis, showing the top 20 signaling pathways.

**Figure 6:**
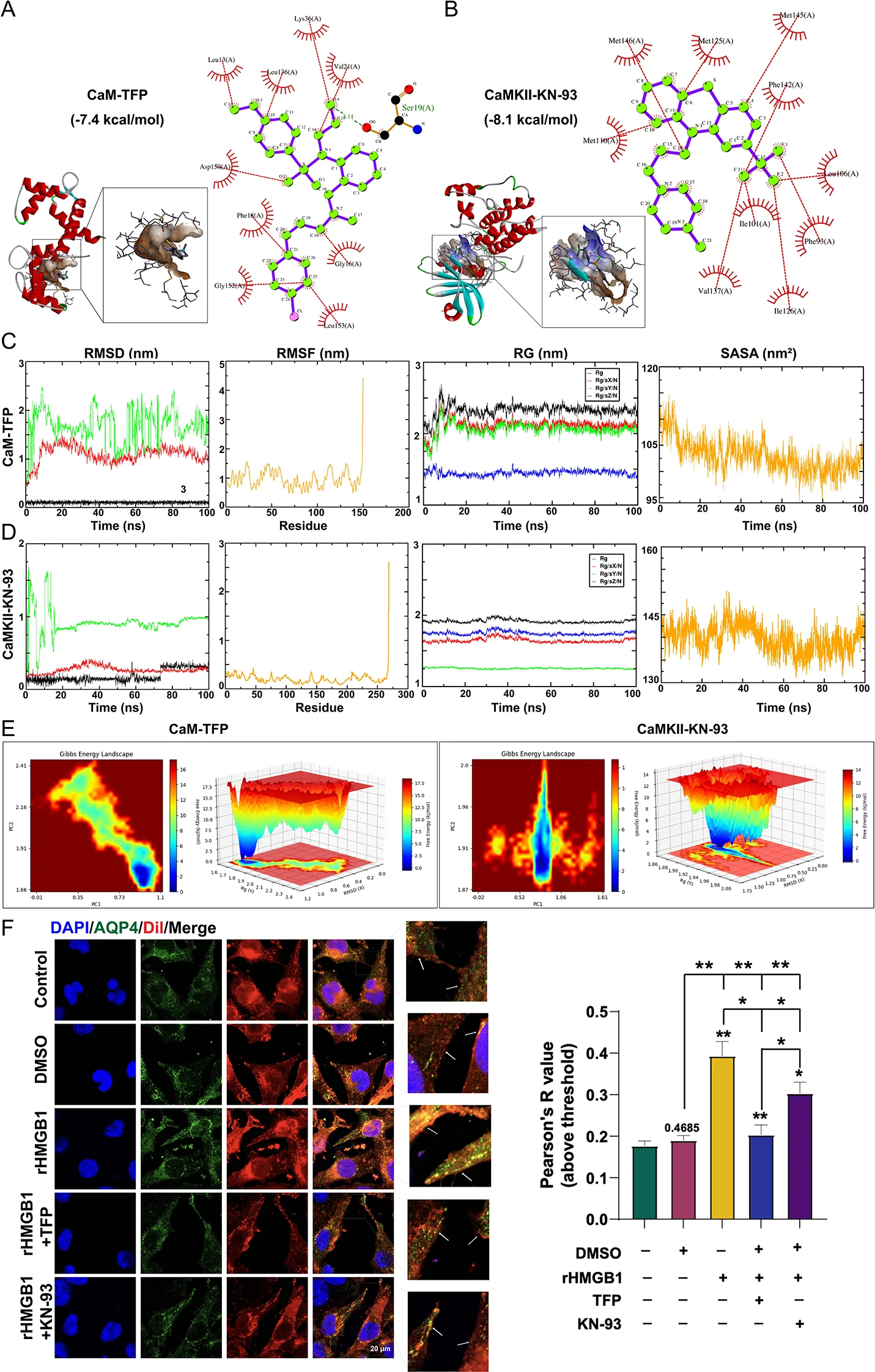
Molecular docking, molecular dynamics simulation, and *in vitro* validation. **(A, B)** Molecular Docking Analysis. **(A)** The binding energy for the CaM-TFP complex is calculated at -7.4 kcal/mol; **(B)** The binding energy for the CaMKII-KN-93 complex is determined to be -8.1 kcal/mol. Left: The three-dimensional structural representation of the complex; middle: a magnified depiction of the binding pocket; right: a two-dimensional interaction schematic. **(C, D)** Molecular Dynamics Simulations Conducted Over 100 ns. **(C)** CaM-TFP; **(D)** CaMKII-KN-93. From left to right: Root Mean Square Deviation (RMSD), Root Mean Square Fluctuation (RMSF), Radius of Gyration (Rg), and Solvent Accessible Surface Area (SASA). **(E)** Free Energy Landscape Exploration. The Gibbs free energy map is derived from Principal Component Analysis (PCA) (left) and is accompanied by a three-dimensional energy surface visualization (right). **(F)** Immunofluorescence Assessment of the Inhibitory Impact of TFP and KN-93 on AQP4 Membrane Translocation. Left: Fluorescence imaging of various treatment groups; right: Quantitative evaluation using the Pearson correlation coefficient (*p < 0.05, **p<0.01).

A calmodulin (CaM) antagonist, trifluoperazine (TFP), has been approved as an antipsychotic by both the US FDA and the UK NICE (30). Using the Jvenn tool, we generated a Venn diagram of shared targets between encephalitis and TFP (**Figure 5A**). A total of 254 overlapping targets were identified (**Figure 6B**; **Supplementary Figure 2**). These targets were analyzed via the STRING database for protein–protein interactions (PPIs) (**Figure 5B**) and visualized via Cytoscape, which revealed calmodulin (CALM1/2/3) (**Figure 5C**).Gene Ontology (GO) analysis (DAVID) of the 254 potential targets revealed the top terms for biological processes (BPs; inflammatory response, protein phosphorylation), cellular components (CCs; membrane, extracellular space), and molecular functions (MFs; protein binding, ATP binding) (**Figure 5D**). KEGG enrichment analysis showed that calcium signaling pathways, cAMP signaling pathway, PI3K-AKT signaling pathway and others were significantly enriched (**Figure 5E**). These results suggesting that the antipsychotic drug trifluoperazine may treat encephalitis by inhibiting calmodulin and reducing inflammation, providing a new direction for future research and medication.

### 3.7 Molecular dynamics simulation and cell membrane AQP4

The molecular docking results indicated that the binding energy between TFP and CaM was -7.4 kcal/mol (**Figure 6A**), whereas the binding energy between KN-93 and CaMKII was -8.1 kcal/mol (**Figure 6B**). Both inhibitors formed stable complexes with their respective target proteins through hydrogen bonding and hydrophobic interactions, with KN-93 demonstrating a stronger binding affinity. The 2D interaction diagrams revealed that both TFP and KN-93 established specific interactions with multiple amino acid residues of their respective target proteins, suggesting good target selectivity. Molecular dynamics simulations of the two complexes were conducted for 100 ns to evaluate their dynamic stability. RMSD analysis demonstrated that both CaM-TFP and CaMKII-KN-93 complexes rapidly reached equilibrium during the simulations, with RMSD values stabilizing at approximately 0.2 nm and 0.25 nm, respectively (**Figures 6C, D**), indicating overall structural stability. RMSF analysis showed that most residues exhibited small fluctuations (<0.3 nm), although the N-terminus of CaM and certain regions of the CaMKII kinase domain displayed higher flexibility. The Rg and SASA curves remained stable throughout the simulations, further confirming the compactness and stability of the complexes’ structures. Free energy landscape diagrams based on principal component analysis (PCA) (**Figure 6E**) demonstrated that the CaM-TFP and CaMKII-KN-93 complexes predominantly occupied a low-energy, stable conformational state during the simulations, with deep and concentrated energy basins, indicating that both inhibitors could effectively stabilize the active conformation of the target proteins and suppress their functional activity. Immunofluorescence experiments (**Figure 6F**, **Supplementary Figure 5**) revealed that, compared to the HMGB1-only treatment group, pretreatment with TFP or KN-93 significantly reduced the expression of AQP4 on the cell membrane (evidenced by decreased green fluorescence intensity). Quantitative analysis demonstrated that HMGB1 induced a significant upregulation of plasma membrane AQP4 expression. Similarly, the AQP4 expression levels in the inhibitor treatment groups were significantly elevated, most notably in the trifluoperazine (TFP) treatment group, indicating that both inhibitors effectively blocked the translocation of AQP4 to the cell membrane by inhibiting the activities of CaM and CaMKII, thereby confirming the biological relevance of the molecular simulation results. While both TFP and KN-93 significantly attenuated HMGB1-induced AQP4 membrane localization, neither fully restored levels to the DMSO control, suggesting that additional signaling pathways may contribute to AQP4 translocation.

### 3.8 TFP alleviates HMGB1-induced BBB dysfunction

**Figure 7:**
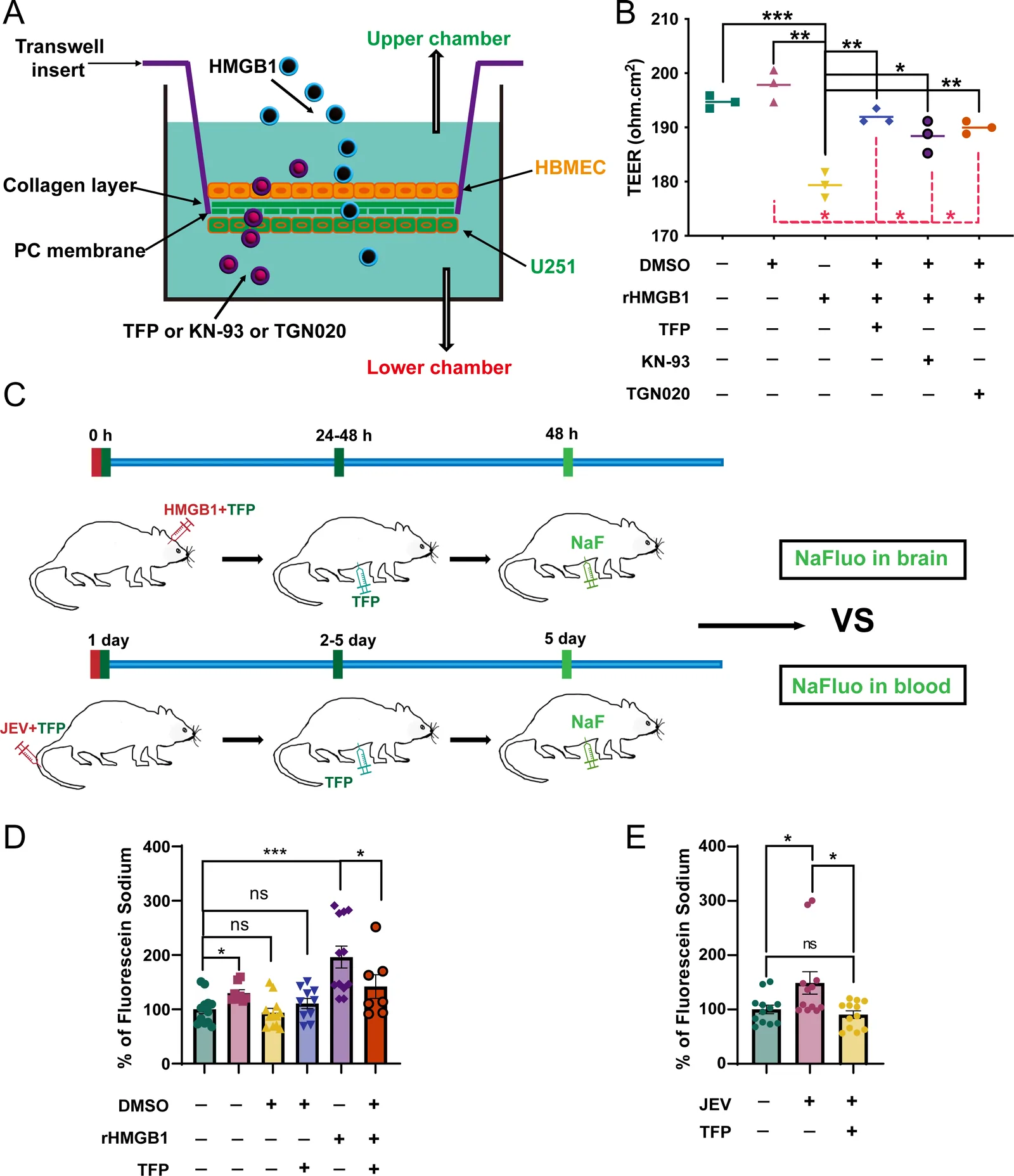
TFP’s role in blood-brain barrier protection. **(A)** An *in vitro* model of the blood-brain barrier was developed and subsequently treated with TFP, KN-93, or TGN020. **(B)** Transendothelial barrier was developed and subsequently treated with TFP, KN-93, or TGN020. **(B)** Transendothelial electrical resistance (TEER) measurements were conducted at 12 hours to evaluate the model’s integrity (n=3). **(C)** A schematic diagram depicts the blood-brain barrier permeability test during HMGB1 and JEV infection *in vivo*. **(D)** An experimental flowchart outlines the assessment of blood-brain barrier integrity in mice administered with NaFluo. **(E)** Recombinant HMGB1 and TFP were intracranially injected into the mouse brain, followed by an intraperitoneal injection of NaFluo after 48 hours. The disruptive effect of HMGB1 on the blood-brain barrier and the protective effect of TFP were evaluated *in vivo* (n≥6). **(E)** During JEV infection, TFP was intracranially injected, and the permeability of the blood-brain barrier to NaFluo was assessed (n≥6). All experiments were repeated at least thrice. Data are expressed as the means ± SEMs. *p < 0.05, **p < 0.01, ***p < 0.001. ns, non significant.

Although HMGB1 promotes AQP4 translocation, the contribution of astrocytic AQP4 to BBB integrity remains unclear. To study the effects of AQP4 on BBB permeability, inhibitors of CaM (TFP), CaMKII (KN-93), and AQP4 (TGN020) were applied to a BBB double-layer model. Consistent with previous results, rHMGB1 significantly decreased the TEER of the BBB (**Figures 7A, B**). Importantly, TFP and KN-93 reversed this reduction in TEER, whereas TGN-020 preserved BBB integrity (**Figures 7A, B**). Thus, pharmacological inhibition of AQP4 translocation or activity attenuates HMGB1-mediated BBB disruption during JEV infection.

Given that AQP4 membrane localization requires direct CaM interaction (30), we evaluated TFP *in vivo*. Intracerebral rHMGB1 injection increased BBB permeability, as assessed by the blood/brain NaFluo fluorescence ratio (**Figures 7C, D**). Although DMSO and TFP alone had no effect, TFP significantly reduced HMGB1-induced BBB leakage (**Figure 7D**). Importantly, TFP administration decreased BBB permeability in JEV-infected mice (**Figure 7E**). These results demonstrate that JEV infection activates the HMGB1–CaM–AQP4 axis.

The elevation of intracellular Ca²^+^ levels regulates AQP4 translocation and activity via CaM, and AQP4 contributes to HMGB1-induced BBB disruption during JEV infection. By blocking this signal transduction via TFP, the CaM antagonist alleviated the BBB dysfunction caused by HMGB1 during JEV infection.

## 4 Discussion

**Figure 8:**
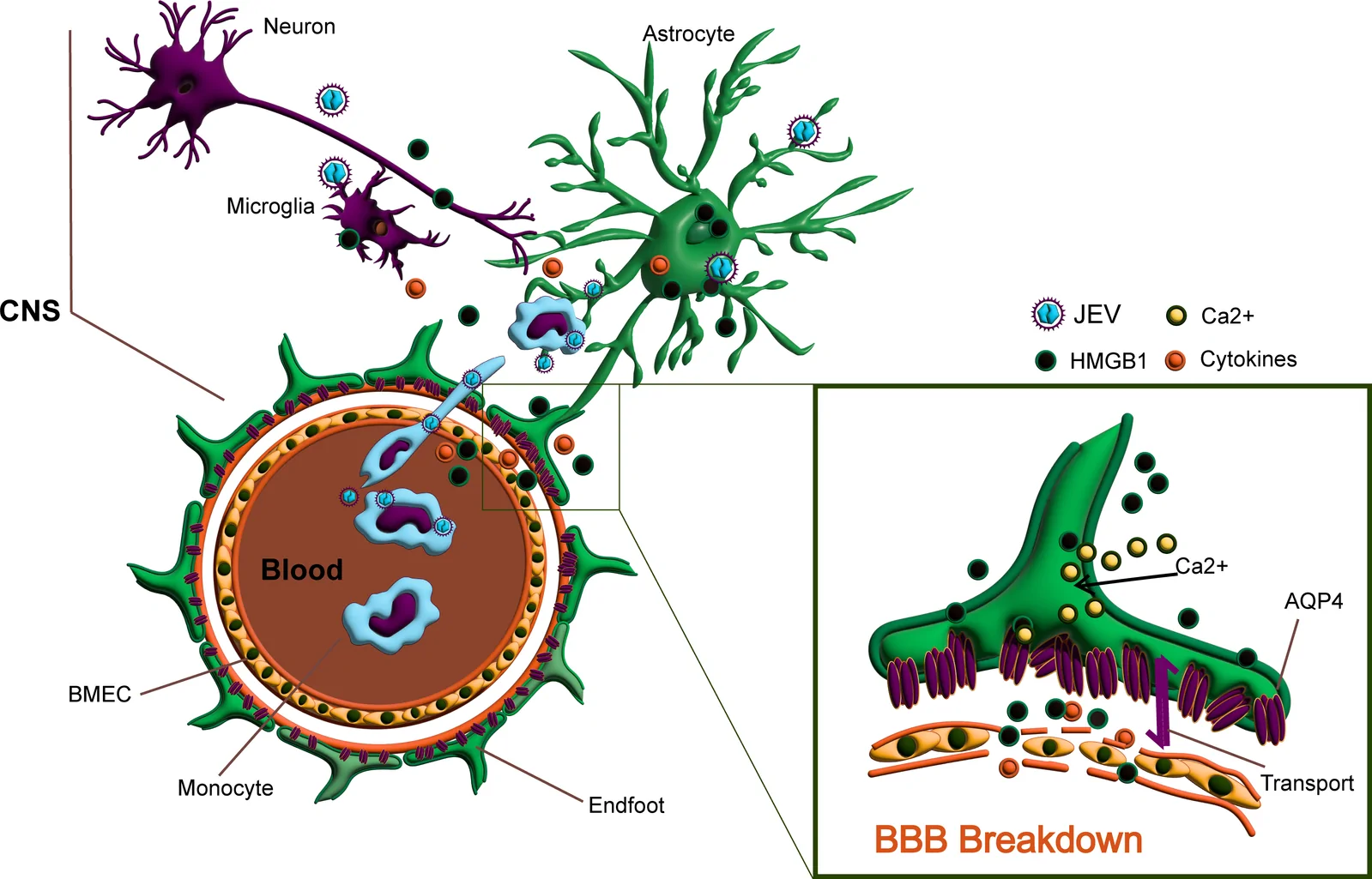
This schematic illustrates the mechanism by which astrocyte-derived HMGB1 facilitates the subcellular translocation of AQP4 and contributes to the disruption of the blood-brain barrier during Japanese encephalitis virus (JEV) infection. The virus infiltrates the central nervous system through the “Trojan horse” mechanism, prompting the release of HMGB1 from astrocytes. Additionally, microglia and neurons may also be involved in the release of HMGB1. The extracellular presence of HMGB1 induces increased intracellular Ca²^+^ fluorescence in astrocytes, thereby activating calcium signaling pathways. This activation leads to the relocation of AQP4 to the cell membrane, which in turn compromises the integrity of the blood-brain barrier. The administration of TFP has been shown to mitigate this effect.

JEV invasion of the CNS activates astrocytes and triggers HMGB1 release, exacerbating BBB permeability. We demonstrated that AQP4 in astrocyte endfeet mediates HMGB1-induced BBB disruption and that inhibition of AQP4 membrane translocation effectively maintains BBB integrity. Furthermore, extracellular HMGB1 functions as a DAMP, establishing a positive feedback loop in astrocytes that amplifies BBB breakdown (**Figure 8**).

While JEV initially enters the CNS via immune cell-mediated “Trojan horse” transport (7), subsequent viral activation of neuroglia induces inflammatory cytokine secretion, which triggers BBB disruption and neuronal damage (6, 8). As a DAMP, extracellular HMGB1 potently amplifies neuroinflammation and BBB destruction (20, 21). Although HMGB1 is ubiquitously expressed, our data specifically implicate astrocyte-derived HMGB1 as a central regulator of BBB integrity, with microglia and neurones serving as potential secondary sources.

Studies have indicated that alarmins (e.g. HMGB1 and S100s) drive disease progression during viral infections (20, 21, 39, 40). Notably, chronic neuroinflammation in neurodegenerative diseases involves HMGB1 release and positive feedback (41). The self-sustaining nature of HMGB1 signaling underscores its therapeutic relevance (42). Although the present study focuses on astrocyte-derived HMGB1, neurons also express HMGB1 receptors (RAGE, TLR2/4) and may contribute to BBB disruption. HMGB1-induced CaM/CaMKII activation and subsequent Nav1.6 hyperactivation could trigger neuronal hyperexcitability (43), indirectly impairing BBB integrity via neurovascular coupling dysfunction. Future studies using astrocyte-specific vs. neuron-specific HMGB1 knockout models will help dissect the relative roles of these cell populations in JEV-induced BBB breakdown. Our *in vitro* BBB model confirmed that HMGB1 disrupted barrier function (**Figures 2F, G**), consistent with reports of HMGB1-mediated ZO-1 degradation and MMP activation (6, 25). *Recombinant* HMGB1 induced significant sodium fluorescein (NaFluo) leakage *in vivo* (**Figure 7D**), validating its role in BBB disruption.

Several CNS diseases, including AD, share common pathological features, such as neuroinflammation and BBB dysfunction (2). To elucidate the role of TFP in BBB disruption, we integrated network pharmacology with GEO database analysis. Network pharmacology was used to predict TFP targets and pathways and verify them with gene expression data from the GEO database to reveal how TFP affects the BBB and AQP4. This approach reveals not only the direct effects of TFP but also the potential indirect effects, providing a foundation for drug repurposing. KEGG analysis highlighted the significant enrichment of calcium-related signaling pathways, including calcium, cAMP, and cGMP-PKG signaling, across multiple CNS diseases, which is consistent with previous studies (35). We used molecular docking and molecular dynamics simulations to confirm the stable TFP–calcium (CaM) binding. As an FDA-approved psychiatric drug, the therapeutic effect of TFP is mediated by its interaction with CaM, thereby inhibiting calcium signaling, which is implicated in the pathogenesis of CNS diseases, suggesting a potential therapeutic strategy for CNS diseases.

Astrocytes, which are essential for the BBB and CNS homeostasis (11, 44), express AQP4 on astrocytic endfeet enveloping brain microvessels (45), suggesting that AQP4 is involved in BBB disruption. AQP4 is closely associated with neuroinflammation and BBB breakdown in multiple disease models (28, 31, 34, 46), which is consistent with our findings of JEV infection. In models of spinal cord injury, cerebral ischemia, and Parkinson’s disease, AQP4 exhibits proinflammatory properties (47). In an experimental autoimmune encephalomyelitis (EAE) model, AQP4 depletion effectively reduces IL-6 production, neuroinflammation, and demyelination. AQP4 deficiency attenuates LPS-induced brain inflammation, alleviating nerve injury and BBB disruption (48). AQP4 was identified as an important participant in HMGB1-mediated BBB disruption.

Notably, the increased of intracellular Ca²^+^ activates calmodulin (CaM), promoting AQP4 membrane translocation and edema (30). Reducing the degree of membrane translocation of AQP4 decreases the degree of brain edema, which is beneficial for neural recovery (30). It is important to note that Fluo-4 is a single-wavelength Ca²^+^ indicator, and these data therefore reflect relative changes in intracellular Ca²^+^-associated fluorescence rather than absolute Ca²^+^ concentrations. While these findings are consistent with a model in which HMGB1-induced Ca²^+^ signaling contributes to AQP4 translocation, further studies using Ca²^+^ chelators or selective Ca²^+^ channel blockers will be required to establish direct mechanistic causality. While TFP protects BBB integrity in JEV-infected mice, we acknowledge its promiscuous pharmacological activity, including D2 receptor and α1A-adrenergic receptor antagonism. The protective effects observed may therefore reflect modulation of the CaM/AQP4 axis, off-target effects on other signaling pathways, or a combination thereof. Future studies using more selective CaM inhibitors or genetic approaches (e.g., astrocyte-specific CaM knockout) will be needed to definitively establish the contribution of CaM inhibition to TFP-mediated BBB protection.

Our investigation revealed that trifluoperazine (TFP) offers a superior advantage over KN-93 in maintaining the integrity of the blood-brain barrier (BBB), primarily because of its distinctive mechanism and multitarget capabilities. TFP directly interacts with pivotal molecules, such as calmodulin (CaM), thereby inhibiting a range of CaM-dependent signaling pathways (30). In contrast, KN-93’s effects are confined to downstream CaMKII, resulting in comparatively limited protective benefits (49). As an FDA-approved pharmaceutical, TFP modulates dopamine and adrenergic receptors and influences neuroinflammatory pathways (30, 50).which supports astrocyte function and preserves BBB integrity, underscoring its potential for clinical application. The primary objective of this study was to identify pharmacological agents capable of mitigating BBB damage caused by JEV infection, with particular emphasis on the significance of TFP. However, the multi-target nature of TFP presents challenges in distinguishing its CaM-specific effects from indirect effects. Future research should investigate the targeted and non-targeted effects of TFP using cell-specific gene knockout models and selective CaM inhibitors. Although both TFP and KN-93 can alleviate BBB damage induced by HMGB1, neither fully restored normal levels, suggesting the involvement of unidentified pathways in BBB injury. In addition to the calcium signal cascade, HMGB1 concurrently activates the TLR4/NF-κB inflammatory pathway, the RAGE/RhoA cytoskeletal regulation pathway, and the MMPs-mediated protein degradation pathway (42, 51–53). The interplay of these parallel signaling pathways results in the compromise of tight junctions and the integrity of the endothelial barrier, which cannot be effectively countered by inhibitors targeting a single calcium signal. As a result, drugs that focus on a single pathway yield only limited therapeutic benefits. Our research highlights the pivotal role of calcium-related signaling and uncovers the synergistic nature of multiple pathways in HMGB1-induced blood-brain barrier (BBB) disruption. This provides a more comprehensive theoretical framework for future studies on combined drug interventions. It should be noted that the BBB exhibits marked regional heterogeneity: white matter endothelia express higher tight junction protein levels than gray matter (54); endothelial transcriptomes differ along the arteriole–capillary–venule axis (55); and fenestrated versus BBB-type endothelia are maintained by distinct signaling pathways (56). We selected the blood-brain barrier (BBB) primarily located in the cerebral cortex of mice as the main research subject. which is also an area where symptoms become prominent following Japanese encephalitis virus infection (**Figure 1A**). In the experiment, anatomically matched coronal sections from age-matched mice were used to ensure the basal state of the blood-brain barrier remained consistent. Future studies will extend this investigation to other brain regions and to regions with distinct barrier properties.

In this study, we built an *in vitro* BBB model and employed inhibitors to determine the role of AQP4 in HMGB1-mediated BBB disruption. This study focuses solely on JEV infection; the role of AQP4 in maintaining blood-brain barrier homeostasis still requires further investigation. Clarify the regulatory mechanisms of AQP4, optimize its targeting strategies, and validate the clinical efficacy of TFP. In addition, TFP, a clinically prescribed drug, has the potential to alleviate JEV-infection-induced BBB disruption, as validated in a JEV-infected mouse model. Targeting astrocytic endfeet AQP4 suppresses BBB disruption and is a promising clinical strategy.

## 5 Conclusion

This study elucidates the critical role of both *in vitro* and *in vivo* conditions. The HMGB1–CaM-APQ4 axis is implicated in Japanese encephalitis virus (JEV)-induced blood-brain barrier (BBB) disruption. Furthermore, we propose trifluoperazine (TFP) as a therapeutic agent that inhibits AQP4 translocation to preserve BBB integrity, representing a novel strategy for the treatment of central nervous system (CNS) diseases.

## Data Availability

The dataset analyzed in this study is publicly available in the NCBI Gene Expression Omnibus (GEO) repository under accession number GSE154002. The permanent direct access URL is: https://www.ncbi.nlm.nih.gov/geo/query/acc.cgi?acc=GSE154002. All other data supporting the findings of this study are included within the article and its **Supplementary Materials**.

## References

[1] Liebner S Dijkhuizen RM Reiss Y Plate KH Agalliu D Constantin G . Functional morphology of the blood-brain barrier in health and disease. Acta Neuropathol. (2018) 135:311–36. doi: 10.1007/s00401-018-1815-1 29411111 PMC6781630

[2] Daneman R Engelhardt B . Brain barriers in health and disease. Neurobiol Dis. (2017) 107:1–3. doi: 10.1016/j.nbd.2017.05.008 28552387

[3] Spindler KR Hsu TH . Viral disruption of the blood-brain barrier. Trends Microbiol. (2012) 20:282–90. doi: 10.1016/j.tim.2012.03.009 22564250 PMC3367119

[4] Haley NJ Richt JA . Classical bovine spongiform encephalopathy and chronic wasting disease: two sides of the prion coin. Anim Dis. (2023) 3:24. doi: 10.1186/s44149-023-00087-738164791

[5] Zhang Y Wang Z Fang Y Zhu Q Fu J Hu S . Therapeutic potential of the neutralizing monoclonal antibody 45G3 against encephalomyocarditis virus. Anim Dis. (2025) 5:1. doi: 10.1186/s44149-024-00154-738164791

[6] Li F Wang Y Yu L Cao S Wang K Yuan J . Viral infection of the central nervous system and neuroinflammation precede blood-brain barrier disruption during Japanese encephalitis virus infection. J Virol. (2015) 89:5602–14. doi: 10.1128/jvi.00143-15 25762733 PMC4442524

[7] Zou SS Zou QC Xiong WJ Cui NY Wang K Liu HX . Brain microvascular endothelial cell-derived HMGB1 facilitates monocyte adhesion and transmigration to promote JEV neuroinvasion. Front Cell Infect Microbiol. (2021) 11:701820. doi: 10.3389/fcimb.2021.701820 34532298 PMC8439198

[8] Sharma KB Vrati S Kalia M . Pathobiology of Japanese encephalitis virus infection. Mol Aspects Med. (2021) 81:100994. doi: 10.1016/j.mam.2021.100994 34274157

[9] Chambers TJ Diamond MS . Pathogenesis of flavivirus encephalitis. Adv Virus Res. (2003) 60:273–342. doi: 10.1016/s0065-3527(03)60008-4 14689697 PMC7202458

[10] Dong S Yu J Guo Z Zhang X Hu R Xie H . Japanese encephalitis virus infection induces PKM2-dependent glycolysis to promote viral replication. Anim Dis. (2025) 5. doi: 10.1186/s44149-025-00199-238164791

[11] Obermeier B Daneman R Ransohoff RM . Development, maintenance and disruption of the blood-brain barrier. Nat Med. (2013) 19:1584–96. doi: 10.1038/nm.3407 24309662 PMC4080800

[12] Langen UH Ayloo S Gu C . Development and cell biology of the blood-brain barrier. Annu Rev Cell Dev Biol. (2019) 35:591–613. doi: 10.1146/annurev-cellbio-100617-062608 31299172 PMC8934576

[13] Giovannoni F Quintana FJ . The role of astrocytes in CNS inflammation. Trends Immunol. (2020) 41:805–19. doi: 10.1016/j.it.2020.07.007 32800705 PMC8284746

[14] Lee HG Wheeler MA Quintana FJ . Function and therapeutic value of astrocytes in neurological diseases. Nat Rev Drug Discov. (2022) 21:339–58. doi: 10.1038/s41573-022-00390-x 35173313 PMC9081171

[15] Ghoshal A Das S Ghosh S Mishra MK Sharma V Koli P . Proinflammatory mediators released by activated microglia induces neuronal death in Japanese encephalitis. Glia. (2007) 55:483–96. doi: 10.1002/glia.20474 17203475

[16] Kuo YC Lu CH . Effect of human astrocytes on the characteristics of human brain-microvascular endothelial cells in the blood-brain barrier. Colloids Surf B Biointerfaces. (2011) 86:225–31. doi: 10.1016/j.colsurfb.2011.04.005 21524890

[17] Nation DA Sweeney MD Montagne A Sagare AP D'Orazio LM Pachicano M . Blood-brain barrier breakdown is an early biomarker of human cognitive dysfunction. Nat Med. (2019) 25:270–6. doi: 10.1038/s41591-018-0297-y 30643288 PMC6367058

[18] Al-Obaidi MMJ Bahadoran A Wang SM Manikam R Raju CS Sekaran SD . Disruption of the blood brain barrier is vital property of neurotropic viral infection of the central nervous system. Acta Virol. (2018) 62:16–27. doi: 10.4149/av_2018_102 29521099

[19] Linnerbauer M Wheeler MA Quintana FJ . Astrocyte crosstalk in CNS inflammation. Neuron. (2020) 108:608–22. doi: 10.1016/j.neuron.2020.08.012 32898475 PMC7704785

[20] Kang R Chen R Zhang Q Hou W Wu S Cao L . HMGB1 in health and disease. Mol Aspects Med. (2014) 40:1–116. doi: 10.1016/j.mam.2014.05.001 25010388 PMC4254084

[21] Bertheloot D Latz E . HMGB1, IL-1alpha, IL-33 and S100 proteins: dual-function alarmins. Cell Mol Immunol. (2017) 14:43–64. doi: 10.1038/cmi.2016.34 27569562 PMC5214941

[22] Sasaki T Liu K Agari T Yasuhara T Morimoto J Okazaki M . Anti-high mobility group box 1 antibody exerts neuroprotection in a rat model of Parkinson's disease. Exp Neurol. (2016) 275:220–31. doi: 10.1016/j.expneurol.2015.11.003 26555088

[23] Okuma Y Liu K Wake H Zhang J Maruo T Date I . Anti-high mobility group box-1 antibody therapy for traumatic brain injury. Ann Neurol. (2012) 72:373–84. doi: 10.1248/yakushi.13-00255-2 22915134

[24] Bailly C Vergoten G . Glycyrrhizin: An alternative drug for the treatment of COVID-19 infection and the associated respiratory syndrome? Pharmacol Ther. (2020) 214:107618. doi: 10.1016/j.pharmthera.2020.107618 32592716 PMC7311916

[25] Festoff BW Sajja RK van Dreden P Cucullo L . HMGB1 and thrombin mediate the blood-brain barrier dysfunction acting as biomarkers of neuroinflammation and progression to neurodegeneration in Alzheimer's disease. J Neuroinflamm. (2016) 13:194. doi: 10.1186/s12974-016-0670-z 27553758 PMC4995775

[26] Thurgur H Pinteaux E . Microglia in the neurovascular unit: Blood-brain barrier-microglia interactions after central nervous system disorders. Neuroscience. (2019) 405:55–67. doi: 10.1016/j.neuroscience.2018.06.046 31007172

[27] Nico B Frigeri A Nicchia GP Quondamatteo F Herken R Errede M . Role of aquaporin-4 water channel in the development and integrity of the blood-brain barrier. J Cell Sci. (2001) 114:1297–307. doi: 10.1242/jcs.114.7.1297 11256996

[28] Genel O Pariante CM Borsini A . The role of AQP4 in the pathogenesis of depression, and possible related mechanisms. Brain Behav Immun. (2021) 98:366–77. doi: 10.1016/j.bbi.2021.08.232 34474133

[29] Wang C Yan M Jiang H Wang Q He S Chen J . Mechanism of aquaporin 4 (AQP 4) up-regulation in rat cerebral edema under hypobaric hypoxia and the preventative effect of puerarin. Life Sci. (2018) 193:270–81. doi: 10.1016/j.lfs.2017.10.021 29054452

[30] Kitchen P Salman MM Halsey AM Clarke-Bland C MacDonald JA Ishida H . Targeting aquaporin-4 subcellular localization to treat central nervous system edema. Cell. (2020) 181:784–99:e719. doi: 10.1016/j.cell.2020.03.037 32413299 PMC7242911

[31] Nagelhus EA Ottersen OP . Physiological roles of aquaporin-4 in brain. Physiol Rev. (2013) 93:1543–62. doi: 10.1152/physrev.00011.2013 24137016 PMC3858210

[32] Fukuda AM Badaut J . Aquaporin 4: a player in cerebral edema and neuroinflammation. J Neuroinflamm. (2012) 9:279. doi: 10.1186/1742-2094-9-279 23270503 PMC3552817

[33] Pan QL Lin FX Liu N Chen RC . The role of aquaporin 4 (AQP4) in spinal cord injury. BioMed Pharmacother. (2022) 145:112384. doi: 10.1016/j.biopha.2021.112384 34915672

[34] Sun L Li M Ma X Feng H Song J Lv C . Inhibition of HMGB1 reduces rat spinal cord astrocytic swelling and AQP4 expression after oxygen-glucose deprivation and reoxygenation via TLR4 and NF-kappaB signaling in an IL-6-dependent manner. J Neuroinflamm. (2017) 14:231. doi: 10.1186/s12974-017-1008-1 29178911 PMC5702193

[35] De Silva MI Gan HK Bardy C . Repurposing trifluoperazine for glioblastoma treatment. Trends Pharmacol Sci. (2025) 46:392–406. doi: 10.1016/j.tips.2025.03.005 40300936

[36] Wang K Wang H Lou W Ma L Li Y Zhang N . IP-10 promotes blood-brain barrier damage by inducing tumor necrosis factor alpha production in Japanese encephalitis. Front Immunol. (2018) 9:1148. doi: 10.3389/fimmu.2018.01148 29910805 PMC5992377

[37] Xian P Hei Y Wang R Wang T Yang J Li J . Mesenchymal stem cell-derived exosomes as a nanotherapeutic agent for amelioration of inflammation-induced astrocyte alterations in mice. Theranostics. (2019) 9:5956–75. doi: 10.7150/thno.33872 31534531 PMC6735367

[38] Abdulmahdi W Patel D Rabadi MM Azar T Jules E Lipphardt M . HMGB1 redox during sepsis. Redox Biol. (2017) 13:600–7. doi: 10.1016/j.redox.2017.08.001 28806702 PMC5554965

[39] Wang S Zhang Y . HMGB1 in inflammation and cancer. J Hematol Oncol. (2020) 13:116. doi: 10.1186/s13045-020-00950-x 32831115 PMC7443612

[40] Fang P Schachner M Shen YQ . HMGB1 in development and diseases of the central nervous system. Mol Neurobiol. (2012) 45:499–506. doi: 10.1007/s12035-012-8264-y 22580958

[41] Paudel YN Shaikh MF Chakraborti A Kumari Y Aledo-Serrano A Aleksovska K . HMGB1: A common biomarker and potential target for TBI, neuroinflammation, epilepsy, and cognitive dysfunction. Front Neurosci. (2018) 12:628. doi: 10.3389/fnins.2018.00628 30271319 PMC6142787

[42] Fan H Tang HB Chen Z Wang HQ Zhang L Jiang Y . Inhibiting HMGB1-RAGE axis prevents pro-inflammatory macrophages/microglia polarization and affords neuroprotection after spinal cord injury. J Neuroinflamm. (2020) 17:295. doi: 10.1186/s12974-020-01973-4 33036632 PMC7547440

[43] Zybura AS Baucum AJ Rush AM Cummins TR Hudmon A . CaMKII enhances voltage-gated sodium channel Nav1.6 activity and neuronal excitability. J Biol Chem. (2020) 295:11845–65. doi: 10.1074/jbc.ra120.014062 32611770 PMC7450116

[44] Segarra M Aburto MR Hefendehl J Acker-Palmer A . Neurovascular interactions in the nervous system. Annu Rev Cell Dev Biol. (2019) 35:615–35. doi: 10.1146/annurev-cellbio-100818-125142 31590587

[45] Graber DJ Levy M Kerr D Wade WF . Neuromyelitis optica pathogenesis and aquaporin 4. J Neuroinflamm. (2008) 5:22. doi: 10.1186/1742-2094-5-22 18510734 PMC2427020

[46] Nicchia GP Nico B Camassa LM Mola MG Loh N Dermietzel R . The role of aquaporin-4 in the blood-brain barrier development and integrity: studies in animal and cell culture models. Neuroscience. (2004) 129:935–46. doi: 10.1016/j.neuroscience.2004.07.055 15561409

[47] Li L Zhang H Verkman AS . Greatly attenuated experimental autoimmune encephalomyelitis in aquaporin-4 knockout mice. BMC Neurosci. (2009) 10:94. doi: 10.1186/1471-2202-10-94 19660138 PMC3152780

[48] Li L Zhang H Varrin-Doyer M Zamvil SS Verkman AS . Proinflammatory role of aquaporin-4 in autoimmune neuroinflammation. FASEB J. (2011) 25:1556–66. doi: 10.1096/fj.10-177279 21257712 PMC3079299

[49] Alphonse N Wanford JJ Voak AA Gay J Venkhaya S Burroughs O . A family of conserved bacterial virulence factors dampens interferon responses by blocking calcium signaling. Cell. (2022) 185:2354:+. doi: 10.1016/j.cell.2022.04.028 35568036 PMC9596379

[50] Wang J Liang M Shang Q Qian HY An R Liu H . Psilocin suppresses methamphetamine-induced hyperlocomotion and acquisition of conditioned place preference via D2R-mediated ERK signaling. CNS Neurosci Ther. (2023) 29:831–41. doi: 10.1111/cns.14054 36627756 PMC9928547

[51] Paudel YN Angelopoulou E Piperi C Othman I Aamir K Shaikh MF . Impact of HMGB1, RAGE, and TLR4 in Alzheimer's disease (AD): From risk factors to therapeutic targeting. Cells. (2020) 9. doi: 10.3390/cells9020383 32046119 PMC7072620

[52] Wang F Ji S Wang M Liu L Li Q Jiang F . HMGB1 promoted P-glycoprotein at the blood-brain barrier in MCAO rats via TLR4/NF-kappaB signaling pathway. Eur J Pharmacol. (2020) 880:173189. doi: 10.1016/j.ejphar.2020.173189 32417325

[53] Chen R Kang R Tang D . The mechanism of HMGB1 secretion and release. Exp Mol Med. (2022) 54:91–102. doi: 10.1038/s12276-022-00736-w 35217834 PMC8894452

[54] Noumbissi ME Galasso B Stins MF . Brain vascular heterogeneity: Implications for disease pathogenesis and design of *in vitro* blood-brain barrier models. Fluids Barriers CNS. (2018) 15:12. doi: 10.1186/s12987-018-0097-2 29688865 PMC5911972

[55] Schaeffer S Iadecola C . Revisiting the neurovascular unit. Nat Neurosci. (2021) 24:1198–209. doi: 10.1038/s41593-021-00904-7 34354283 PMC9462551

[56] Matsuoka RL Buck LD Vajrala KP Quick RE Card OA . Historical and current perspectives on blood endothelial cell heterogeneity in the brain. Cell Mol Life Sci. (2022) 79:372. doi: 10.1007/s00018-022-04403-1 35726097 PMC9209386

