# Correction: Astrocyte-derived HMGB1 compromises the integrity of the blood-brain barrier through the CaM/CaMKII/AQP4 pathway and the protective function of trifluoperazine

**DOI:** 10.3389/fimmu.2026.1987139

**Published:** 2026-10-05

**Authors:** Song-Song Zou, Li-Li Chen, Min Cui

**Affiliations:** 1 Key Laboratory of Exploitation and Study of Distinctive Plants of Sichuan Provincial Education Department, Sichuan University of Arts and Science, Dazhou, China; 2 State Key Laboratory of Agricultural Microbiology, College of Veterinary Medicine, Huazhong Agricultural University, Wuhan, China; 3 Key Laboratory of Preventive Veterinary Medicine in Hubei Province, The Cooperative Innovation Centre for Sustainable Pig Production, Hubei Jiangxia Laboratory, Wuhan, Hubei, China; 4 Key Laboratory of Development of Veterinary Diagnostic Products, Ministry of Agriculture of the People’s Republic of China, Wuhan, Hubei, China; 5 International Research Centre for Animal Disease, Ministry of Science and Technology of the People’s Republic of China, Wuhan, Hubei, China; 6 Dazhou Traditional Chinese Medicine (TCM) Research and Development Centre (R&D), Dazhou, China; 7 Dazhou TCM Industry Incubation Center, Dazhou, China

**Keywords:** blood-brain barrier, HMGB1, JEV, network pharmacology, trifluoperazine

There was a mistake in **Figure 6E** as published. **Figure 6E** contained an inadvertent image error arising from figure assembly.

**Figure 6:**
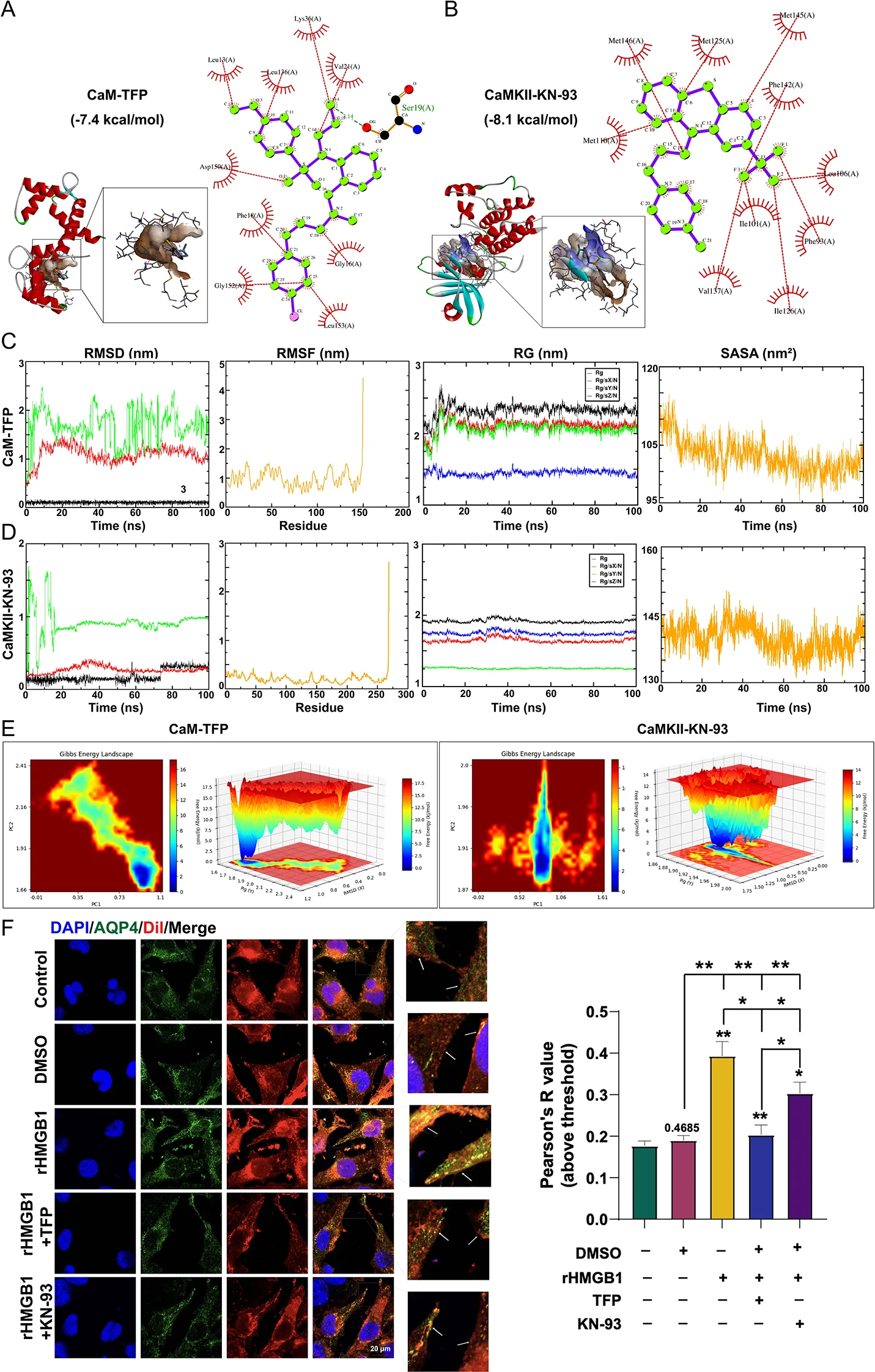
Molecular docking, molecular dynamics simulation, and in vitro validation. **(A, B)** Molecular Docking Analysis. **(A)** The binding energy for the CaM-TFP complex is calculated at -7.4 kcal/mol; **(B)** The binding energy for the CaMKII-KN-93 complex is determined to be -8.1 kcal/mol. Left: The three-dimensional structural representation of the complex; middle: a magnified depiction of the binding pocket; right: a two-dimensional interaction schematic. **(C, D)** Molecular Dynamics Simulations Conducted Over 100 ns. **(C)** CaM-TFP; **(D)** CaMKII-KN-93. From left to right: Root Mean Square Deviation (RMSD), Root Mean Square Fluctuation (RMSF), Radius of Gyration (Rg), and Solvent Accessible Surface Area (SASA). **(E)** Free Energy Landscape Exploration. The Gibbs free energy map is derived from Principal Component Analysis (PCA) (left) and is accompanied by a three-dimensional energy surface visualization (right). **(F)** Immunofluorescence Assessment of the Inhibitory Impact of TFP and KN-93 on AQP4 Membrane Translocation. Left: Fluorescence imaging of various treatment groups; right: Quantitative evaluation using the Pearson correlation coefficient (*p < 0.05, **p<0.01).

The corrected figure appears below.

The original version of this article has been updated.

