# Supplementary material for: Astrocyte-derived HMGB1 compromises the integrity of the blood-brain barrier through the CaM/CaMKII/AQP4 pathway and the protective function of trifluoperazine
Source: Front Immunol. 2026 Jun 23;17:1852083. doi: 10.3389/fimmu.2026.1852083 (PMC13337370; doi:10.3389/fimmu.2026.1852083)
Supplement: Supplementary file 1 [file SupplementaryFile1.zip › Supplementary files/Supplementary Figure S1.DOCX]

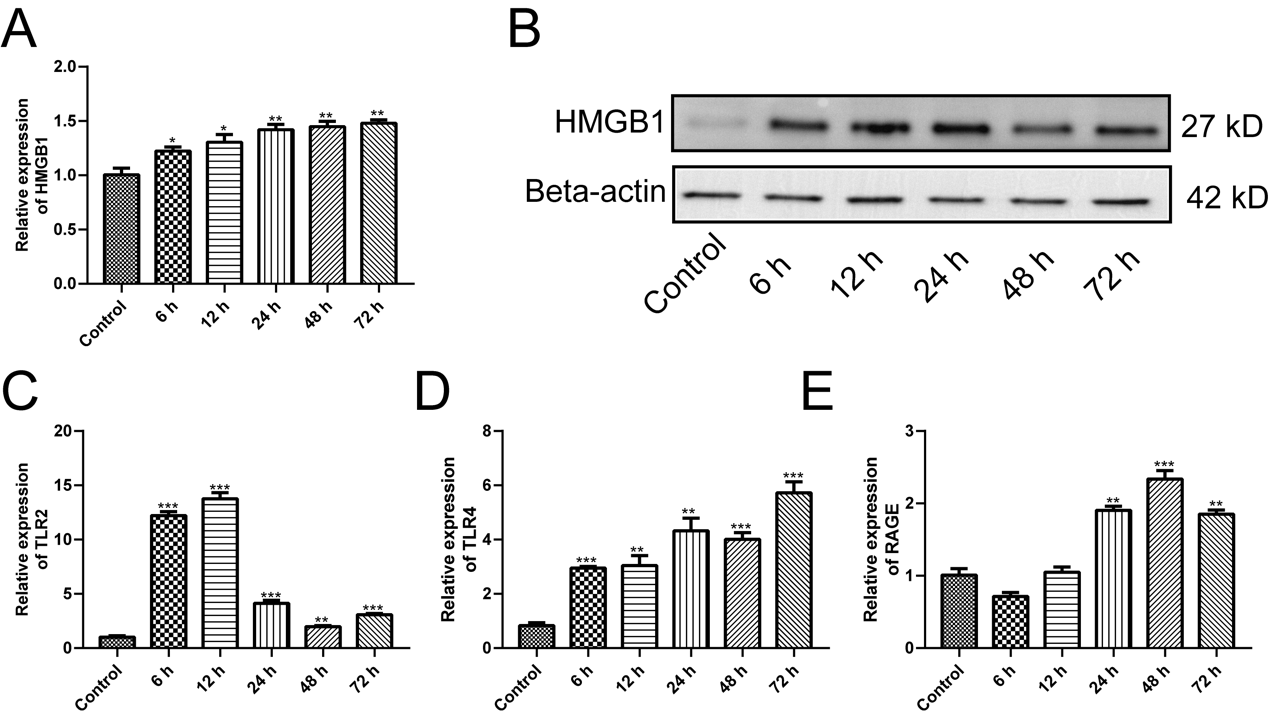


Supplementary Figure 1 Detection of HMGB1 expression in rHMGB1-treated primary astrocytes. Real-time PCR (A) and Western blot (B) were used to measure the mRNA and protein levels of HMGB1 in primary astrocytes after rHMGB1 treated. Real-time PCR were used to measure the mRNA levels of RAGE and TLR2/4 in primary astrocytes after rHMGB1 treatment (C-E). All experiments were repeated at least three times. These data were expressed with the means ± SEM. *p < 0.05, **p < 0.01, and ***p < 0.001.


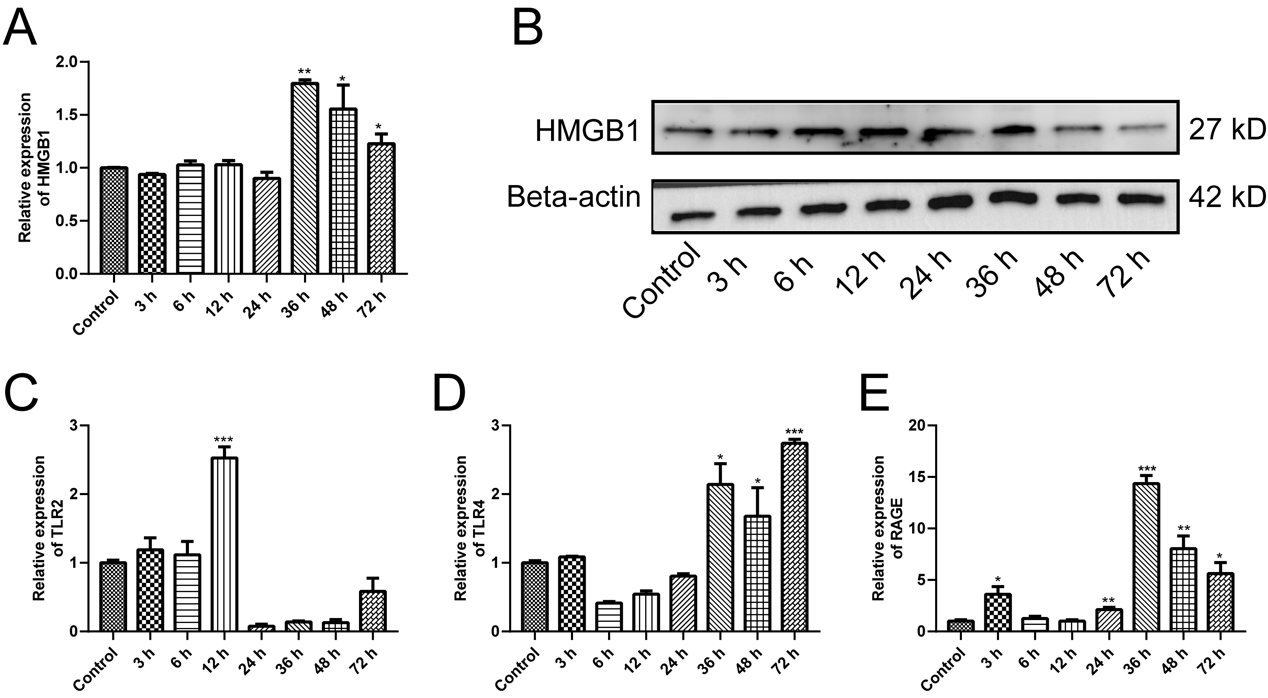


Supplementary Figure 2 Detection of HMGB1 expression in rHMGB1-treated U251 cells. Real-time PCR (A) and Western blot (B) were used to measure the mRNA and protein levels of HMGB1 in U251 cells after rHMGB1 treated. Real-time PCR were used to measure the mRNA levels of RAGE and TLR2/4 in U251 cells after rHMGB1 treatment (C-E). All experiments were repeated at least three times. These data were expressed with the means ± SEM. *p < 0.05, **p < 0.01, and ***p < 0.001.


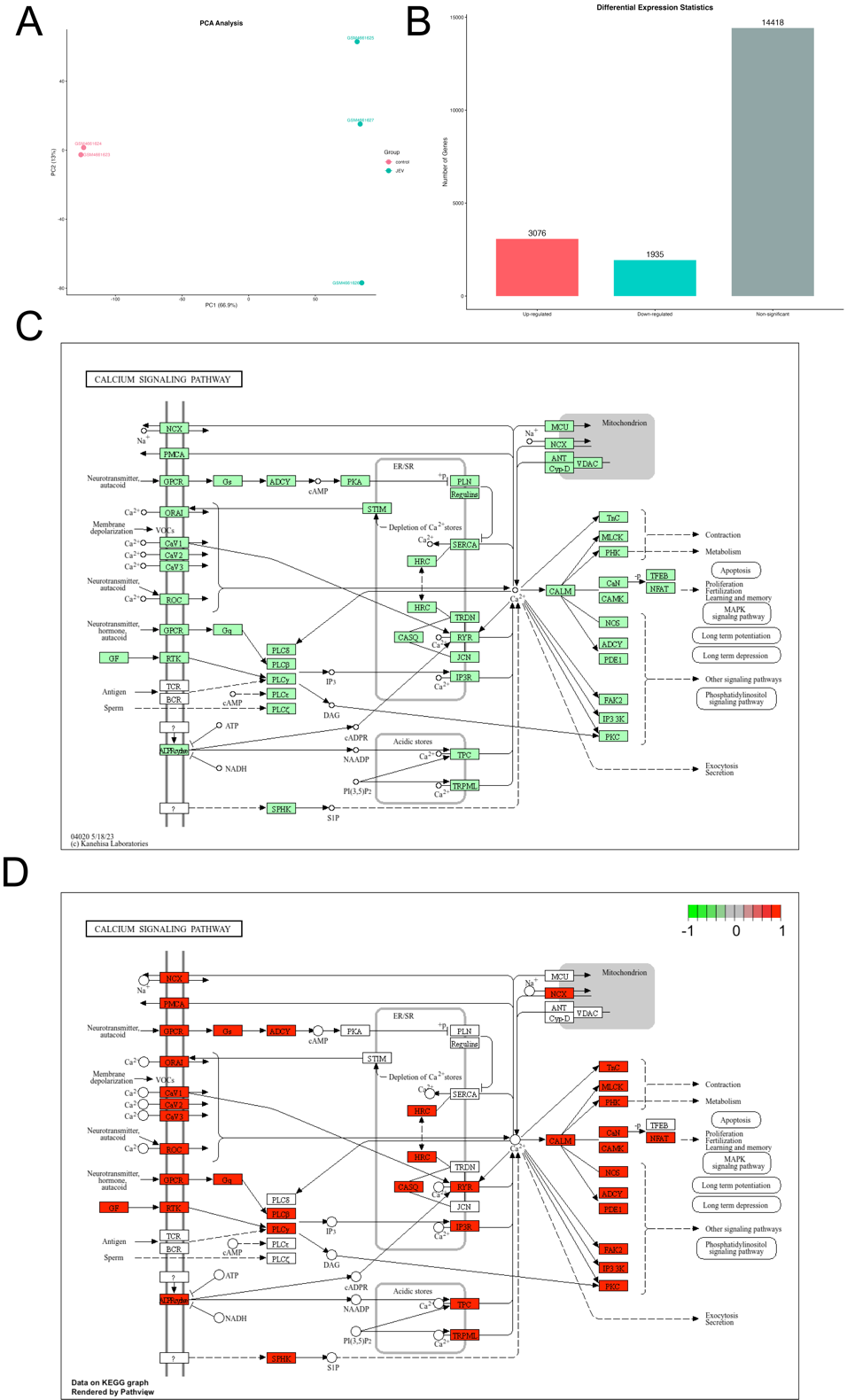


Supplementary Figure 3 Transcriptome analysis of differentially expressed genes (DEGs) and functional enrichment between JEV-infected and control. (A) Bar plot showing the number of up-regulated, down-regulated, and non-differentially expressed genes. (B) MA plot of RNA-seq data, evaluating the overall expression distribution and differential analysis reliability. Green boxes (C) and red boxes (D) indicate genes with altered expression in the pathway. The figures show the distribution and regulatory relationships of differentially expressed genes within this pathway, revealing their mechanism of action in biological processes.


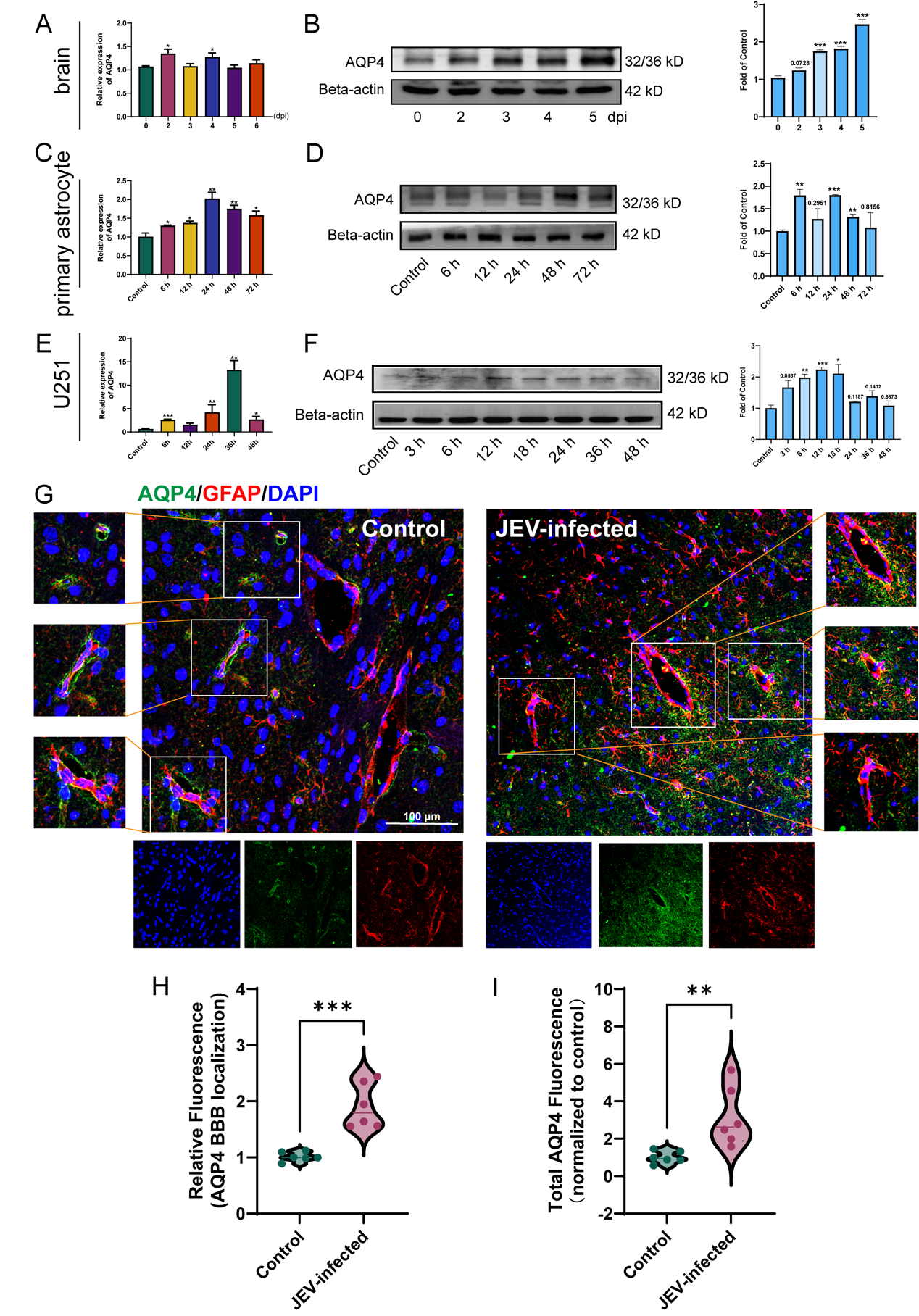


Supplementary Figure 4 Detection of AQP4 in the mouse brain and the association of HMGB1 and AQP4 during JEV-infected. Real-time PCR (A) and Western blotting (B) were used to measure the mRNA and protein levels of AQP4 in mouse brain tissue. Real-time PCR (C) and Western blotting (D) were employed to quantify AQP4 mRNA and protein levels in JEV-infected primary astrocytes. Real-time PCR (E) and Western blotting (F) were also used to measure AQP4 mRNA and protein expression in JEV-infected U251 cells. (G) Immunofluorescence images of JEV-infected mouse brain tissue stained for AQP4 (green), GFAP (red), and nuclei (blue) to visualize protein localization. The scale bar for (G) is 100 μm. (H) Quantification of images shows changes in AQP4 BSCB localization. (I) Quantification of images shows changes in total AQP4 expression. All experiments were repeated at least three times. Statistical analysis of Western blot data is shown in the blue histogram. Data are expressed as the means ± SEMs. Statistical significance is denoted as *p < 0.05, **p < 0.01, and ***p < 0.001.


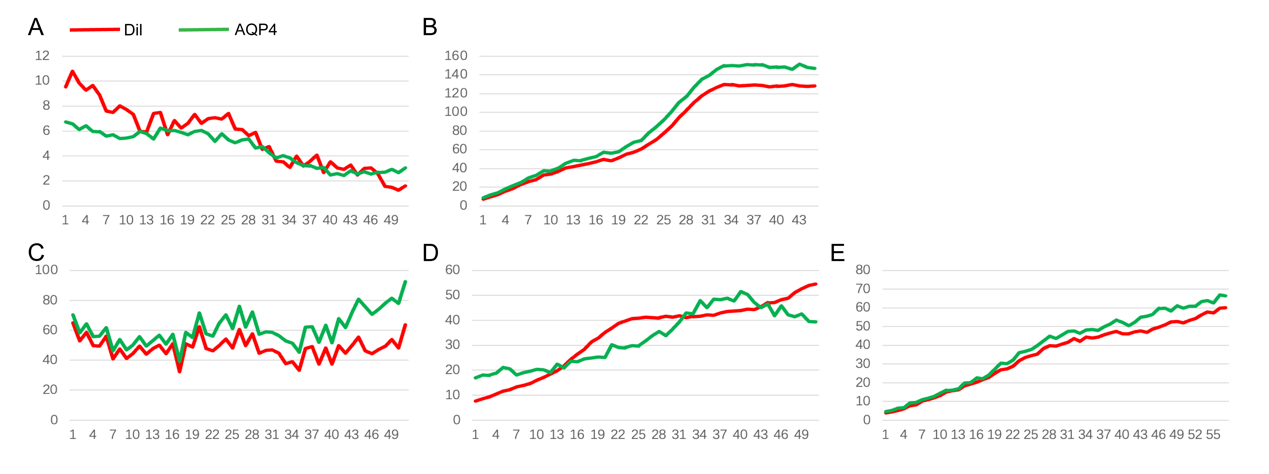


Supplementary Figure 5 The colocalization between AQP4 and cell membrane (Dil). A qualitative representation of fluorescence co-localization of Control (A), DMSO (B), rHMGB1(C), TFP-rHMGB1 (D), KN-93-rHMGB1 (E), which can be achieved by using the gray scale values of fluorescence with scribe distance obtained by the Plot Profile tool in ImageJ.
