# Supplementary figures and images for: Astrocyte-derived HMGB1 compromises the integrity of the blood-brain barrier through the CaM/CaMKII/AQP4 pathway and the protective function of trifluoperazine
Source: Front Immunol. 2026 Jun 23;17:1852083. doi: 10.3389/fimmu.2026.1852083 (PMC13337370; doi:10.3389/fimmu.2026.1852083)

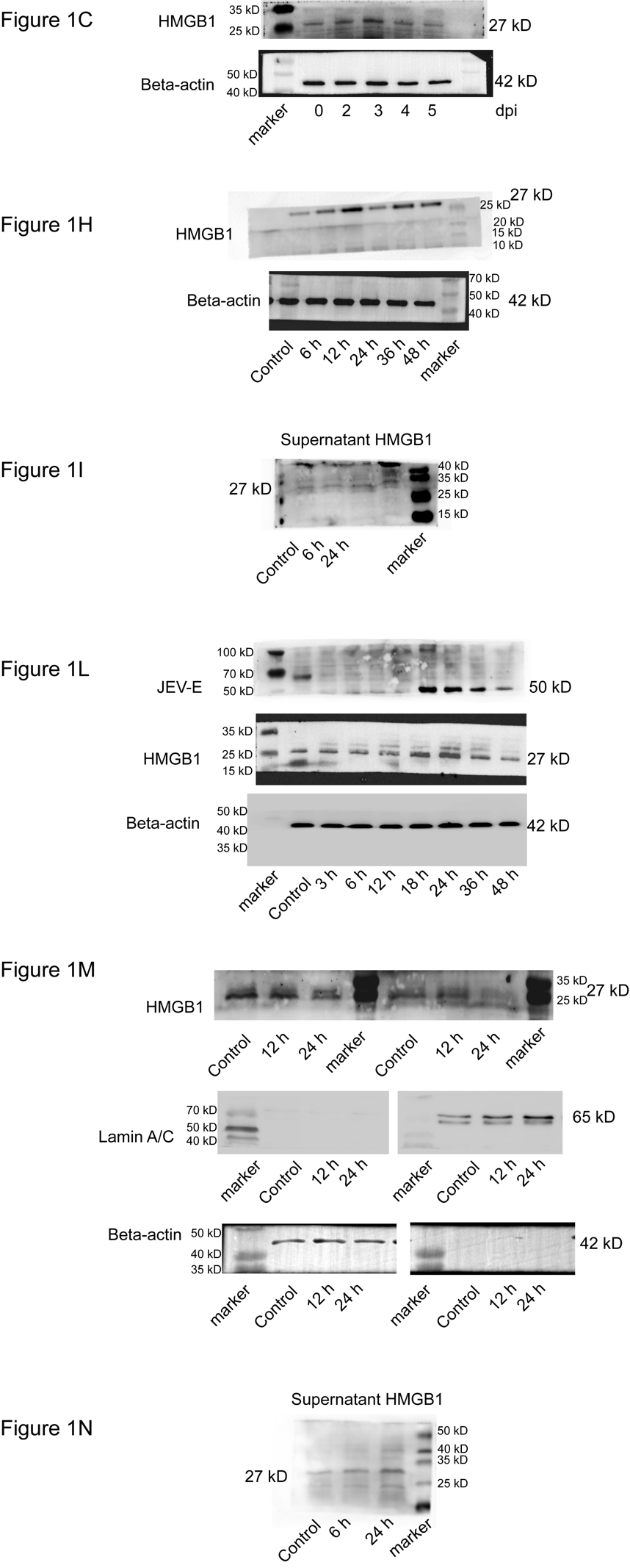


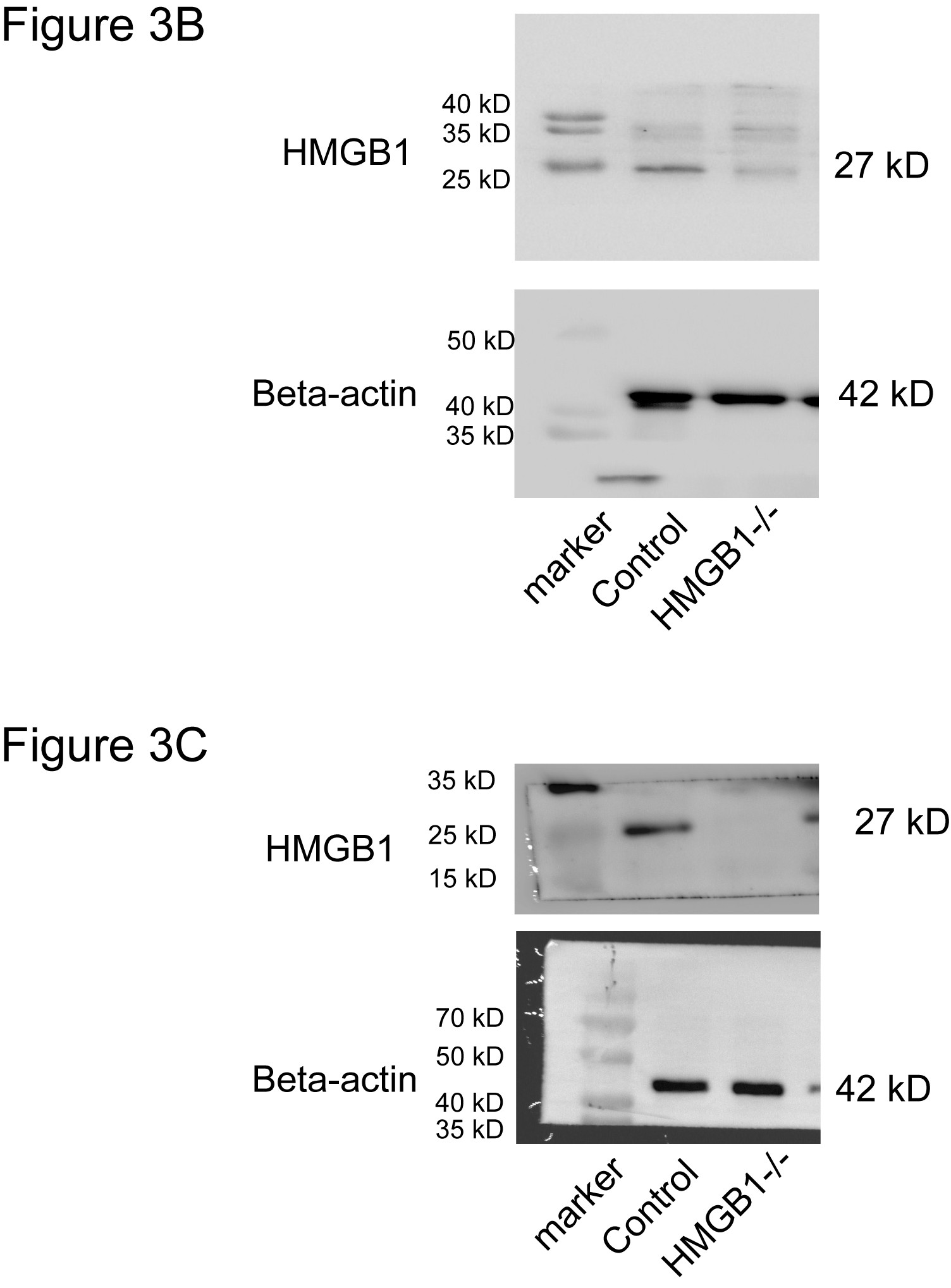


Figure 3C

Figure 3B


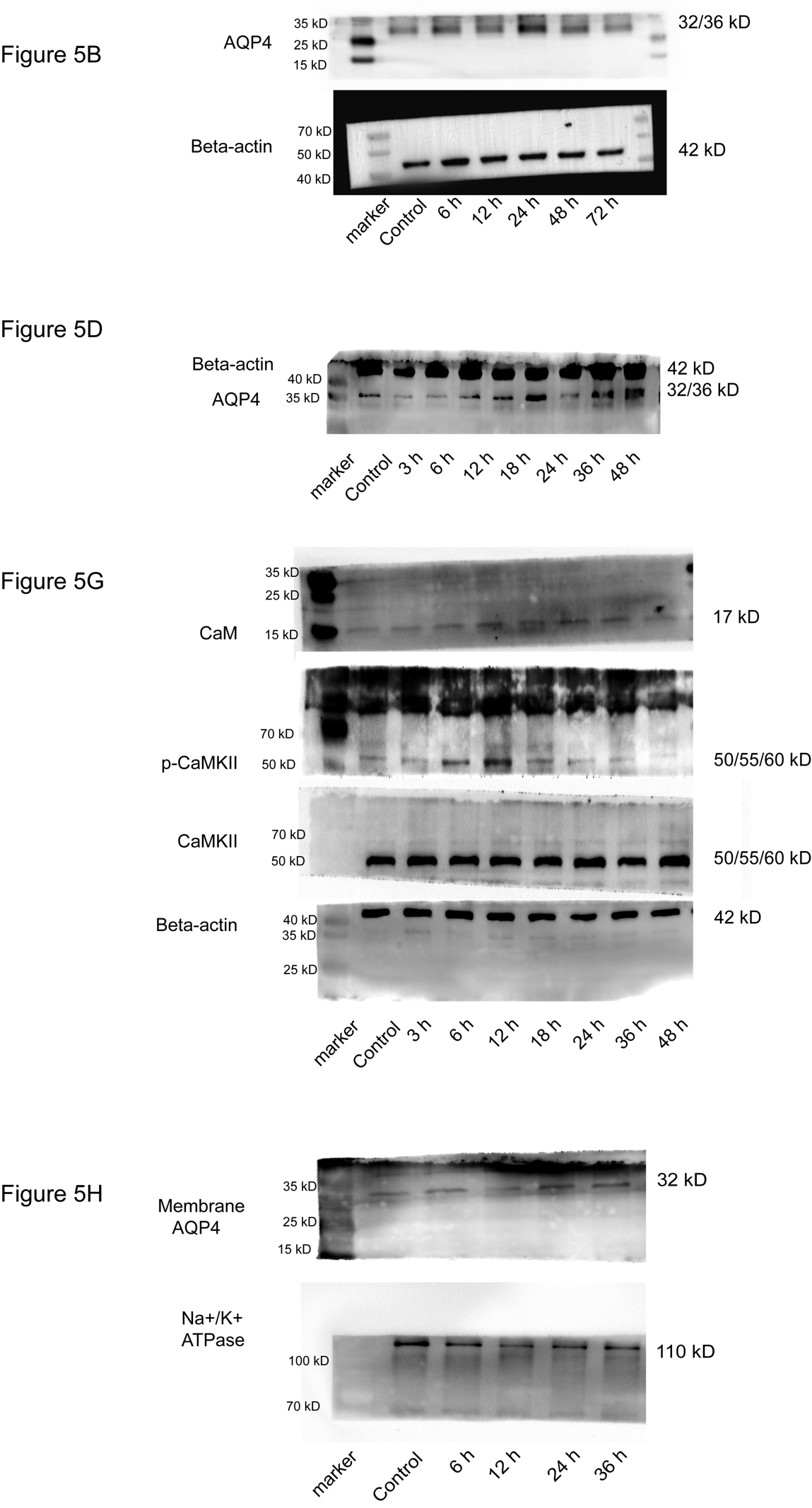


Figure 4C

Figure 4H

Figure 4F


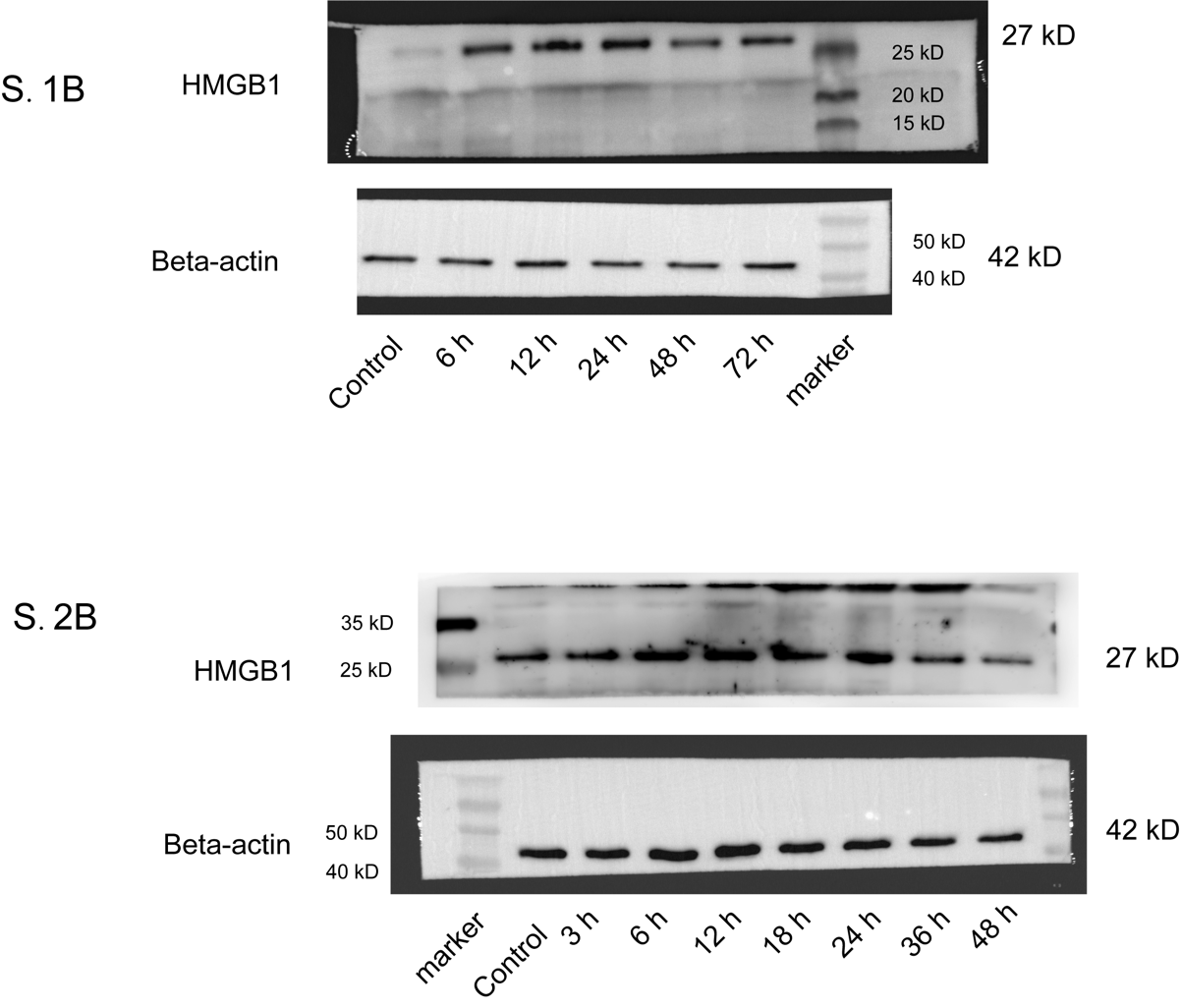


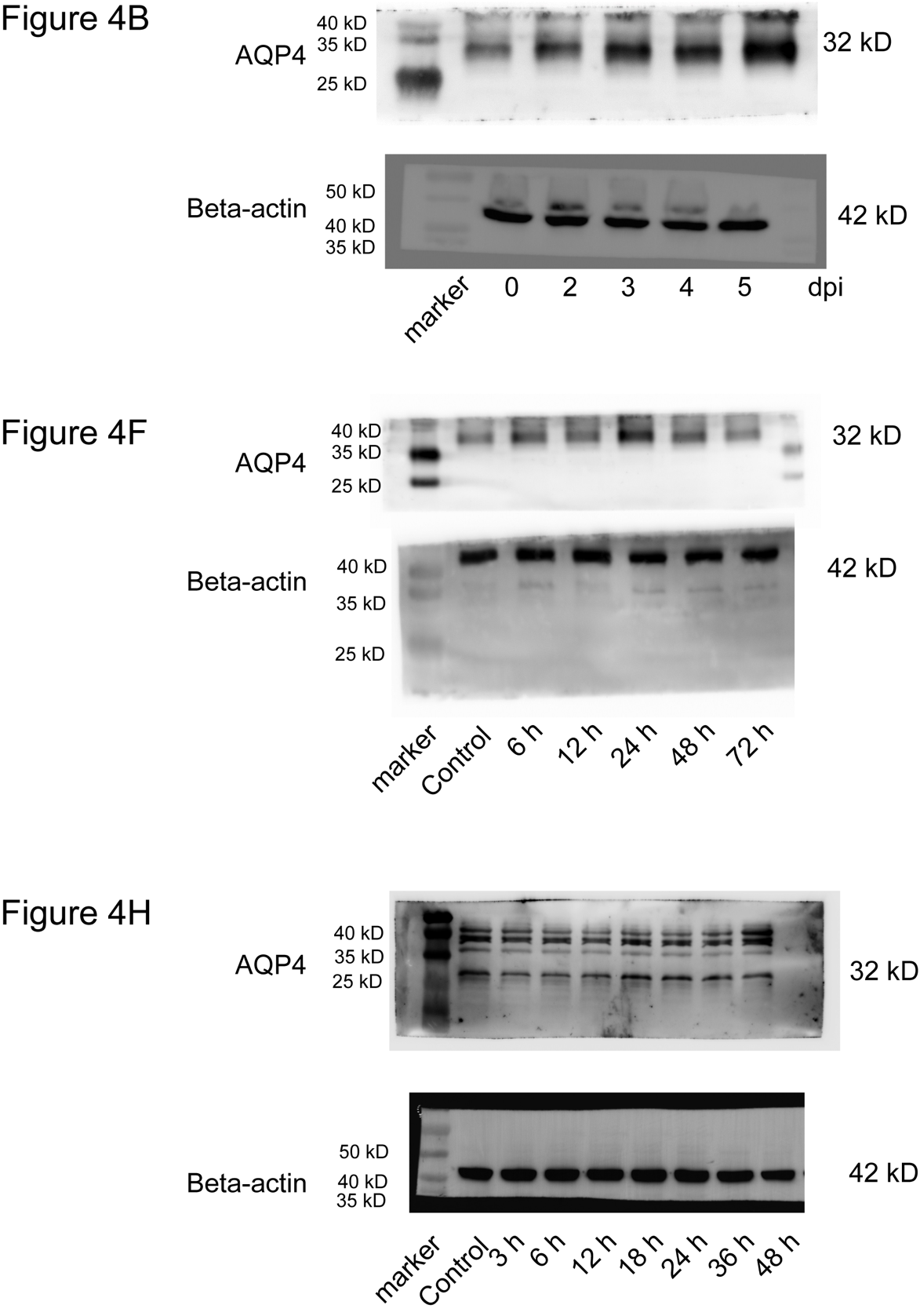


S. 3F

S. 3D

S. 3B

Supplement: Supplementary file 1 [file SupplementaryFile1.zip › Supplementary files/Raw Western blot (WB) images.DOCX]
